# Engineering to Improve Mechanical Properties of Nanocellulose Hydrogels from Aloe Vera Bagasse and Banana Pseudostem for Biomedical Applications

**DOI:** 10.3390/polym17121642

**Published:** 2025-06-13

**Authors:** Rocío Hernández-Leal, Ángeles Iveth Licona-Aguilar, Miguel Antonio Domínguez-Crespo, Esther Ramírez-Meneses, Adela Eugenia Rodríguez-Salazar, Carlos Juárez-Balderas, Silvia Beatriz Brachetti-Sibaja, Aidé Minerva Torres-Huerta

**Affiliations:** 1 New Materials Department, Instituto Tecnológico de Ciudad Madero, Tecnológico Nacional de México, Ciudad Madero C.P. 89440, Tamaulipas, Mexico; rocio_hleal@hotmail.com; 2 Biotechnology Department, Unidad Profesional Interdisciplinaria de Ingeniería-Palenque, Instituto Politécnico Nacional, Nueva Esperanza C.P. 29960, Chiapas, Mexico; aliconaa@ipn.mx; 3 Nanostructured Materials Department, Unidad Profesional Interdisciplinaria de Ingeniería Hidalgo, Instituto Politécnico Nacional, San Agustín Tlaxiaca C.P. 42162, Hidalgo, Mexico; mdominguezc@ipn.mx; 4 Department of Chemical, Industrial and Food Engineering, Universidad Iberoamericana, Ciudad de México C.P. 01219, Mexico; esther.ramirez@ibero.mx; 5 Technological Innovation, Instituto Politécnico Nacional, CICATA Querétaro, Santiago de Querétaro C.P. 76090, Querétaro, Mexico; aerodriguez@ipn.mx; 6 Department of Engineering Studies for Innovation, Universidad Iberoamericana, Lomas de Santa Fe, Ciudad de México C.P. 01219, Mexico; carlos.juarez@ibero.mx

**Keywords:** agricultural waste, nanocrystalline cellulose, hydrogels, mechanical properties, swelling capability, biomedical applications

## Abstract

This work explores the synthesis of biomass-waste-derived cellulose nanocrystal hydrogel from aloe vera bagasse (AVB) and banana pseudostem (BPS). A wide variety of synthesis parameters such as acid concentration (45 wt.% and 55 wt.%), temperatures in the process of 25, 40, 45 and 50 °C, and reaction times of 30 and 60 min were analyzed during the acid hydrolysis to evaluate changes in the morphology, crystallinity, swelling, degradation temperature, and mechanical properties. The parameters that most influenced the crystallinity were the temperature and reaction time, showing good characteristics such as percentage crystallinity (89.66% for nanocellulose from C_45_t_30_T_50_ up to 97.58% for CNC-BPS C_55_t_30_T_50_), and crystal size (from 23.40 to 68.31 nm), which was worth considering for hydrogel synthesis. Cellulose nanocrystalline hydrogels from both biomass wastes can modify the crystallinity for tailored high-end engineering and biomedical applications, although using BPS obtained the best overall performance; also, properties such as swelling capability at *pH* = 4 of 225.39% for hydrogel C_55_t_30_T_25_ (H7), porosity (60.77 ± 2.60%) for C_45_t_60_T_40_ (H6), and gel % (86.60 ± 2.62%) for C_55_t_60_T_50_ (H8) were found. The mechanical test revealed a tensile strength at maximum load of 707.67 kPa (hydrogel H6) and 644.17 kPa (hydrogel H8), which are properties conferred by the CNC from BPS. Overall, CNC from BPS is recommended as a reinforcement for hydrogel synthesis due to its good mechanical properties and functionals, making it a promising material for biomedical applications.

## 1. Introduction

In recent years, the demand for manufacturing new materials from inexhaustible and sustainable resources has increased considerably amidst global climate change, contributing to the search for green alternatives [1,2,3,4,5,6]. However, the global amount of agricultural, forestry, and industrial wastes generated by various processes has had a negative environmental impact on soil, water, and air, mainly due to improper disposal [7,8,9,10,11]. According to the 2024 report by the United Nations (UN) Environment Programme, food loss and waste collectively impact the global economy by approximately USD 1 trillion annually. This figure accounts not only for the food itself, but also for the energy, labor, and water used in its production. Of this waste, around 45% is of organic origin. In this context, the UN also estimates that agricultural operations generate approximately 0.14 trillion metric tons of biomass waste annually, and this could increase by up to 60% by 2050. This increase is projected to occur alongside a global population growth of 33% between 2015 and 2050, reaching 9.7 billion people [12,13]. In response to this environmental challenge, increasing attention has been given to the use of renewable polymeric materials derived from agricultural by-products. Among these, cellulose fibers have attracted significant research interest due to their biocompatibility, biodegradability, renewability, and non-toxicity, making them a promising raw material for various industries, including textiles, food, pharmaceuticals, water treatment, cosmetics, energy production, and biomedicine [14,15,16,17,18,19]. The growing demand for lignocellulosic-based materials in these applications has driven research into alternatives for extracting the main structural components of biomass. The chemical composition of biomass is primarily cellulose, hemicellulose, and lignin; the percentage of each component depends on the extraction source and processing method [20,21]. Specifically, cellulose is an abundant and renewable organic material. Composites derived from nano- or micro-sized cellulose are widely used in the development of new biodegradable materials that provide environmentally friendly alternatives and meet various technological needs [22,23,24]. There are three categories of nano-sized cellulose: cellulose nanocrystals (CNCs) [25], nanofibrillated or microfibrillated cellulose (NFC or MFC) [26], and bacterial nanocellulose (BNC) [27]. In addition, several cellulose crystalline forms can be found in nature, such as cellulose I, II, III, and IV. Cellulose I exhibits the strongest mechanical properties and has a parallel chain orientation compared to cellulose II, which has an antiparallel chain configuration. Cellulose III and IV can be obtained after specific treatments of cellulose I and II [28]. Common biomasses that have been used as a source of cellulose are sugarcane bagasse [29], corn cobs [30], coconut fibers [31], coffee husks [32], tomato peels [33], and banana peels [34], as well as paper-based waste such as used cardboard boxes [35], old newspapers [36], office papers waste [37], and disposable cups [38], all of which have emerged as viable renewable sources for cellulose isolation with wide-ranging potential applications [39,40,41,42,43,44]. For this reason, various methods have been used to extract cellulose, such as mechanical and chemical treatments, with chemical methods generally being more effective. To expand cellulose functionality, recent research has focused on hydrogel manufacturing. The hydrophilic functional groups contained in cellulose, such as hydroxyl (-OH), carboxyl (-COOH), amide (-CONH_2_), and sulfonic (SO_3_H), enable the production of hydrogel materials with exceptional water absorption and swelling properties, allowing for interaction with anionic surfactants through dipole mechanisms [45,46,47,48,49]. These hydrogels have properties similar to those of biological tissues, and could, therefore, be used in pharmaceutical and biomedical fields [43,50,51]. Thus, through freeze drying or 3D printing, hydrogel materials can be transformed into advanced materials with tailored properties for biomedical applications [52,53].

An important parameter to evaluate in cellulose-based hydrogel production is the crystallinity of the cellulose due to the fact that this can modify the final properties. Aloe vera bagasse (AVB) and banana pseudostem (BPS) biomass are among the underexplored candidate materials for cellulose isolation and for modulating properties for diverse applications [54,55,56]. AVB is a waste generated during the extraction of aloe vera gel. The bark or bagasse is bluish green in color and represents between 20 and 30% of the total weight of the plant, containing around 57% cellulose [57]. On the other hand, BPS consists of concentric layers of leaf sheaths. This residue is produced during the cultivation and cutting of bananas. During this process, a large amount of waste is generated, which, in turn, contains between 63.60% and 68.80% cellulose [58,59].

In our previous work, an initial analysis was reported that served as a crucial basis for experimentation on cellulose extraction from three different agro-industrial wastes. That analysis identified limitations and aspects that were not sufficiently addressed to answer certain research questions [60]. Continuing with this development, this study aims to determine the effect of crystallinity on swelling properties, gel fraction, porosity, and mechanical properties, as well as their potential application in wound healing. In this context, the current work addresses the discovery and evidence scope in the optimization of cellulose extraction and hydrogel synthesis, optimizing parameters such as time, concentration, and industrial waste selection to allow for complete and reproducible experimentation. Additionally, previous publications have focused on the synthesis of cellulose-based hydrogels, reporting that the efficiency of hydrogels is influenced by several factors, with pore size being a critical determinant. In this sense, our experimentation seeks a balance by improving the mechanical properties of the hydrogel through the addition of nanocellulose, guaranteeing the integrity of the hydrogel matrix through the crosslinking process, and controlling the pore size allowed for wound healing applications [61,62].

For the aforementioned reasons, the goal of this study is to evaluate different synthesis parameters during the extraction of cellulose from AVB and BPS waste to determine the influence of crystallinity on the overall performance of cellulose-based hydrogels. To achieve this purpose, changes were made to each step of the treatment process, delignification (ethanol/toluene reflux), cellulose isolation (sodium chlorite solution 100 mL, 2 wt.%), and concentrated acetic acid (1 mL), obtaining nanocrystalline cellulose. The evaluated conditions to control the crystallinity in the last step were two concentrations of acid hydrolysis (45 wt.% and 55 wt.%), different temperatures during the process (25, 40, 45, and 50 °C), and reaction times of 30 and 60 min. It is important to highlight that the hydrogels were manufactured only with the nanocrystalline cellulose samples that displayed the best chemical and structural properties. A 2:1 ratio of cellulose and commercial medium molecular weight chitosan was mixed during the synthesis. In particular, changes in the functional groups, crystallinity index, swelling capacity, thermal stability, and mechanical properties of the as-prepared hydrogels were studied to evaluate their biomedical potential.

## 2. Materials and Methods

### 2.1. Raw Materials and Their Pretreatment

The raw materials, aloe vera bagasse (AVB) and banana pseudostem (BPS), were obtained from plant residues in the metropolitan area of Tampico, Madero, and Altamira Tamaulipas, Mexico (22°17′47″ N 97°52′32″ W). The following reagents were also used: ethanol (C_2_H_6_O, Fermont, Monterrey, Mexico, 99.90% purity); toluene (C_6_H_5_CH_3_, Fermont 99.90% purity); sodium chlorite (NaClO_2_, Sigma Aldrich, Darmstadt, Germany, 80.00% purity); acetic acid (CH_3_COOH, Sigma Aldrich 99.80% purity); sodium hydroxide (NaOH, Sigma Aldrich, 97% purity); sulfuric acid (H_2_SO_4_, JT Baker, Allentown, PA, USA, 98.30% purity); chitosan (Sigma Aldrich, medium molecular weight); dialysis membrane MD25/34/44/55/77 mm; citric acid (Sigma Aldrich, 99.50% purity); and distilled water (Quimitron, Diadema, Brazil). The aloe vera leaves were washed, and a horizontal cut was made at the base of the leaf, which was left to rest for 10 h to eliminate the aloin; thereafter, the gel was extracted. The residual aloe vera bagasse was crushed in a conventional blender and washed several times to eliminate any remaining gel. Then, the material was dried at 90 °C for 24 h. On the other hand, the banana pseudostem (*Musa paradisiaca*) was washed, cut into small pieces, ground in a conventional blender, and then dried at 105 °C for 48 h. After washing and drying, the fibers were ground to a particle size of 500 μm using an analytical mill (Cyclotec CT 293, FOSS, México City, Mexico).

### 2.2. Lignin and Hemicellulose Removal

For the extraction of fats, sugars, chlorophyll, residual pigments, and fat-soluble vitamins, the Soxhlet method was applied, considering the conditions previously reported [63]. Delignification consisted of refluxing a 1:1 ethanol/toluene mixture at 110 °C for 8 h. Then, the fibers were washed with distilled water at 60 °C to remove residual compounds. Subsequently, 3 g of the delignified sample (holocellulose) was placed in a solution of sodium chlorite (100 mL, 2 wt.%) and acetic acid (1 mL) at 60 °C for 2 h with constant stirring. Afterward, the fibrous samples were washed, dried, and added to NaOH (100 mL, 10 wt.%) solution for 3 h while maintaining constant stirring. Finally, the obtained sample (pure cellulose) was filtered, washed with distilled water until a neutral *pH* was obtained, and then dried at 60 °C.

### 2.3. Nanocellulose Isolation

**Figure 1:**
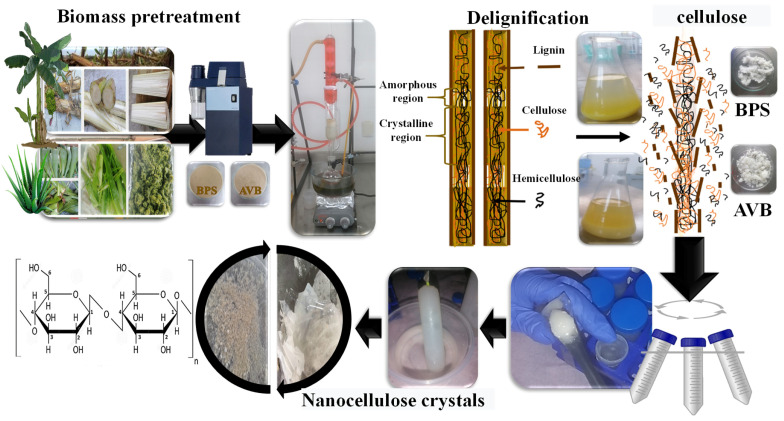
Extraction process of crystalline nanocellulose from banana pseudostem and aloe vera bagasse.

The isolation procedure of the pretreated cellulose samples was carried out by acid hydrolysis using two different concentrations (45 wt.% and 55 wt.%) of H_2_SO_4_ solution at different temperatures (25 °C, 40 °C, 45 °C, and 50 °C) and times of treatment (30 and 60 min). It is important to mention that other acid concentrations, ranging from 25 wt.% to 60 wt.%, were evaluated, but only the concentrations of 45 wt.% and 55 wt.% displayed the most relevant results. Initially, 1.5 g of the previously treated samples were placed in contact with 100 mL of the H_2_SO_4_ solution, applying the temperature and time parameters. The hydrolyzed nanocellulose was centrifuged at 5000 rpm for 20 min through two cycles. The obtained supernatant was removed and poured into a dialysis membrane to remove residual particles and obtain a *pH* close to 7 by washing with distilled water (Figure 1).

### 2.4. Synthesis of Nanocellulose/Chitosan Hydrogels

Chitosan was used for the synthesis of hydrogels. The procedure consisted of the following steps: (i) 1 g of chitosan was dissolved in 2 wt.% acetic acid solution (50 mL) and stirred for 24 h; (ii) 0.50 g of nanocellulose was dissolved in 50 mL of citric acid (0.50 M) until completely dissolved; and (iii) after that time, both samples were mixed, stirred for 24 h, and then poured into silicon molds and dried at 45 °C.

### 2.5. Characterization Techniques

#### 2.5.1. Fourier Transform Infrared Spectroscopy (FTIR)

FTIR analyses were performed to identify the functional groups of the raw materials, extracted cellulose, and synthesized hydrogels using a spectrometer (Perkin Elmer Spectrum 100 FT-IR) within the range of 4000 to 500 cm^−1^.

To confirm the energy and distance hydrogen bonds, the common previously reported equation was used [64,65,66].

(1) $$E_{H}=\frac{1}{k}\frac{\left(v_{o}-v\right)}{v_{o}}$$

In this formula, $v_{o}$ is the standard frequency of the OH groups at 3650 cm^−1^, v is the stretching frequency of the bonded OH groups, and k is obtained from the constant $\frac{1}{k}=2.65\times 10^{2}\;$ in kJ. Similarly, hydrogen bond distances were calculated using the Sederholm equation [67].

(2) $$\Delta v\;\left({\mathrm{cm}}^{-1}\right)=4.43\times 10^{3}\left(2.84-R\right)$$

In this case, Δv is the monomeric OH stretching frequency $\left(v_{o}-v\right)$ located at 3600 cm^−1^ and *R* is the hydrogen bond distance.

This study was complemented with a second-derivative spectra analysis, assigning up to five bands to different hydrogen-bonded vibrations, analyzing the region from 3000 to 3700 cm^−1^. All the peaks were analyzed based on their areas, and a result was considered as good when *r^2^* was greater than 0.99.

#### 2.5.2. Nanocellulose Yield

The yield of the obtained nanocellulose was calculated by the following equation:

(3) $$Yield\;\left(\%\right)=\frac{W_{initial}-W_{final}}{W_{initial}}$$

where $Yield\;\left(\%\right)$ is the percentage of nanocellulose obtained, $W_{initial}$ is the initial weight, and $W_{final}$ corresponds to the final weight.

#### 2.5.3. X-Ray Diffraction (XRD)

The crystalline structures of the raw materials, cellulose, and nanocellulose were identified using an X-ray diffractometer (XRD), with *Cu K_α_* radiation (*λ* = 0.15405 nm), in the 2*θ* range of 6 to 40° at a scan rate of 1° min^−1^. The crystallinity index was calculated using the Segal method, according to the following equation.

(4) $$CI\;\left(\%\right)=\left[\frac{Amorphous\;signal}{Crystalline\;Signal\;}\right]\times 100$$

In the formula, the amorphous signal corresponds to 18° 2*θ* cellulose I or 16° 2*θ* cellulose II and maximum intensity.

The crystallite size was estimated using the Scherrer equation, which relates the width of the signals to the size of the cellulose crystals, using the value of the spherical particles as a shape reference:

(5) $$L=\frac{k\lambda }{\beta *cos\theta \;}$$

where L is the crystallite size (nm), k is the spherical particle value (0.9), *λ* is the X-ray wavelength (Å), β is the full width at half maximum, and *θ* is the XRD angular position.

#### 2.5.4. Dynamic Light Scattering (DLS)

The hydrodynamic particle size of nanocellulose was analyzed using a Litesizer 500 (Dynamic Light Scattering, Particle Analyzer, Anton Paar), equipped with a single-frequency laser diode with a wavelength of 658 nm. For these analyses, the samples (100 µL) were dispersed in distilled water (10 mL), then three dilutions were prepared and sonicated for 60 min. This dilution was used to evaluate the nanocellulose behavior based on Brownian motion.

#### 2.5.5. Scanning Electron Microscopy (SEM)

The morphological features of the materials after each applied treatment were observed using a scanning electron microscope (SEM, JEOL JSM-6O10LA) operated at an accelerating voltage of 20 kV with backscattered electrons and at different magnifications.

#### 2.5.6. Determination of Gel Percentage

The gel percentage was determined as follows: the hydrogels were dried in an oven at 40 °C until a constant weight was achieved; then, they were hydrated in distilled water for 24 h and subsequently dried again at 40 °C until a constant weight was obtained. The percentage was calculated by the ratio of the dry hydrogel weight before and after being immersed in water [68]:

(6) $$Gel\;percentage\;\left(\%\right)=\frac{H_{1}-H_{2}}{H_{1}}\times 100$$

where $H_{1}$ corresponds to the dry hydrogel at a constant weight and $H_{2}$ is the hydrated hydrogel at a constant weight.

#### 2.5.7. Swelling Test

The swelling behavior was analyzed in a phosphate-buffered saline solution (Cl^−^, Na^+^ and K^+^ ions) to simulate the extracellular fluid of mammals. The swelling determination was performed following the procedure described in previous reports [69]. A hydrogel sample with dimensions of 1 × 1 cm was weighed in its dry state. The hydrogel was then immersed in a phosphate buffer solution with varying *pH* values of 4, 7, and 10 at room temperature. Afterward, the swollen hydrogels were placed on adsorbent paper to remove excess moisture from the solution, and the weight of the swollen hydrogel was recorded at various time intervals (3, 6, 12, and 24 h). Finally, the swelling ratio was calculated using Equation (7):

(7) $$\mathrm{Swelling}\;\mathrm{ratio}\;\left(\%\right)=\frac{H_{s}-H_{d}}{H_{d}}\times 100$$

where $H_{s}$ is the weight of the swollen hydrogel and $H_{d}$ corresponds to the dry hydrogel samples.

#### 2.5.8. Thermogravimetric Analysis

The thermal degradation behavior of cellulose hydrogels was investigated using a TGA instrument (SDT Q600 V20.9 Build 20). The applied temperature range was from 30 °C to 500 °C under an inert atmosphere (nitrogen gas) with a heating rate of 10 °C min^−1^.

#### 2.5.9. Mechanical Test

The mechanical properties of the as-prepared hydrogels, along with a commercial hydrogel used as a reference, were evaluated through tension tests, generating stress–strain curves. The relevant tensile properties considered for the evaluation of these materials were tensile strength (stress at maximum load) and strain at maximum load. It is important to highlight that, for this analysis, hydrogels were prepared as films, and the hydrogel specimens used in this test were strips with an average width and thickness of 13.2 mm and 1.14 mm, respectively, and an average gage length of 15.3 mm. The test was carried out at a test speed of 3 mm/min until fracture. An INSTRON 3365 universal testing machine and Bluehill 2 version 2.17 Universal materials testing software were used. The elongation and load data were transferred to OriginPro 9.0 software to generate the stress vs. strain curves for each specimen. Stress (*σ*) was computed as force per unit area, *σ* = *F*/*A*_0_, where *F* is the force or load and *A_0_* is the initial cross-sectional area of the hydrogel specimen. Because the hydrogel undergoes significant deformation during testing, the cross-sectional area decreases as the hydrogel specimen is stretched. The strain (*ε*) was computed as *ε* = Δ*L*/*L*_0_, where *L*_0_ is the gage length or initial length and Δ*L* is the change in length (*L*-*L*_0_) or elongation. The ultimate tensile stress, or tensile strength, is defined as the maximum stress before failure or the stress at maximum load, while the ultimate tensile strain is defined as the strain at maximum load. The percent elongation at break, which is a measure of ductility, was determined as %*EL* = [(*L_f_* − *L*_0_)/*L*_0_] × 100, where *L*_0_ is the initial length and *L_f_* is the final length at break.

#### 2.5.10. Porosity

The porosity of all hydrogels was determined by SEM analysis using ImageJ version 2.14.0/1.54p software. The measurement consisted of determining the total area occupied by the pores in the hydrogel as well as the total area occupied by the hydrogel. The percentage of porosity was determined using the following equation.

(8) $$Porosity\;\left(\%\right)=\frac{Pore\;area}{Total\;sample\;area}\times 100$$

#### 2.5.11. Thermogravimetric Analysis

The thermal degradation behavior of cellulose hydrogels was investigated using a TGA instrument (SDT Q600 V20.9 Build 20). The applied temperature was from 30 °C to 500 °C under an inert atmosphere (nitrogen gas) with a heating rate of 10 °C min^−1^.

## 3. Results and Discussion

### 3.1. Fourier Transform Infrared Spectroscopy

**Figure 2:**
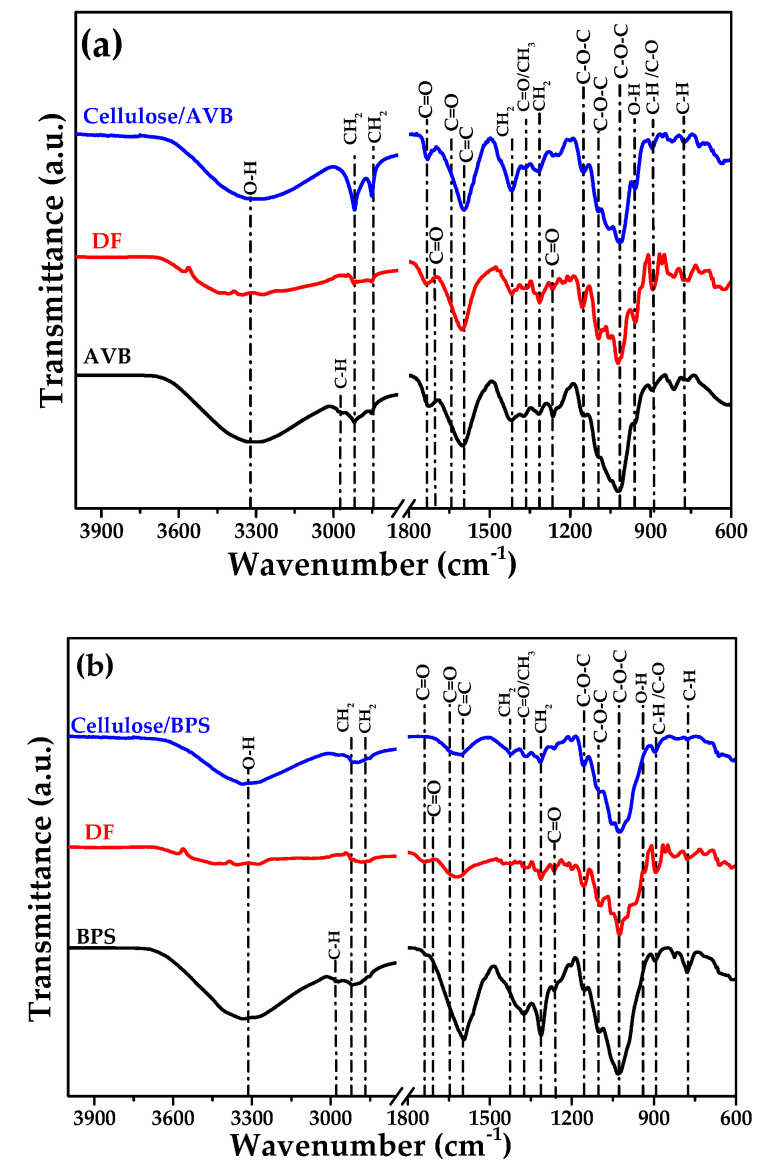
FTIR transmittance spectra of (**a**) aloe vera bagasse (AVB) and (**b**) banana pseudostem (BPS) with a break in the *X*-axis between 3750 and 1800 cm^−1^.

The FT-IR transmittance spectra of the raw materials AVB, BPS, and cellulose are shown in Figure 2a,b. The most important changes in the spectra can be observed in two regions; the first region is located around 2800 cm^−1^ to 3300 cm^−1^ and 800 cm^−1^ to 1700 cm^−1^.

In the FT-IR spectra of AVB, the broad transmittance band at ~3300 cm^−1^ corresponds to the stretching vibration of the hydrogen-bonded hydroxyl group in the cellulosic molecules due to water adsorption (Figure 2a). Peaks at the wavenumbers of 2920 cm^−1^ and 2850 cm^−1^ are characteristic of C-H stretching vibrations in CH_3_ and CH_2_ groups in cellulose, respectively [70,71]. At 1650 cm^−1^ and 1600 cm^−1^, typical vibrations of carbonyl groups and aromatic rings in cellulose, respectively, were observed. The band at 1417 cm^−1^ corresponds to the deformation vibration of the C-H group in the glucose unit, while bending vibrations of C=O and CH_3_ groups corresponding to the cellulose amorphous zone are located at 1366 cm^−1^. At 1313 cm^−1^, the CH_2_ cellulose vibration can be observed. The asymmetric and symmetric stretching of C-O-C in cellulose and hemicellulose is also observed at 1154 cm^−1^. Also, the C-O-C and C-H stretching vibrations of the cellulose component and the β-glycosidic linkage of the glucose ring of cellulose were detected at 1015 cm^−1^, whereas the out-of-plane O-H deformation of carboxylic acid can be found at 955 cm^−1^. The peak at 900 cm^−1^ correlates with the C-H and C-O stretching peaks associated with cellulose linkages.

To verify the removal of lignin, hemicellulose, and other components, the bands located at ~2972 cm^−1^, ~1717 cm^−1^, ~1257 cm^−1^, 1108 cm^−1^, and 826 cm^−1^, related to the vibrations of the methyl-methylene group, carboxyl-stretching unconjugated ketones/carbonyl groups, syringyl ring breathing with CO stretching and the C-O-C stretching vibration of glycosidic bonds from xylans, and the out-of-plane C-H bending of lignin, respectively, should gradually decrease. A reduction in the signals of these bands is observed, but they do not disappear, suggesting that some lignin and hemicellulose components remain at this stage.

On the contrary, the FT-IR spectra of BPS can be observed in Figure 2b. The characteristic vibration bands of cellulose are located at 825 cm^−1^ (C-H), 900 cm^−1^ (C-H/C-O), 940 cm^−1^ (O-H), 1027 cm^−1^ (C-O-C), 1099 cm^−1^ (C-O-C), and 1157 cm^−1^ (C-O-C) [54]. It is evident that all bands are observed in the spectra obtained from both sources, with a slight wavenumber shift, indicating that, although the molecular structure of cellulose remains unchanged after acid hydrolysis treatment, the efficiency of the cellulose isolation depends on the source. It is clear that, in using BPS as a source, some bands disappear, indicating that components such as hemicellulose and lignin were removed from the fibers after chemical treatment, which causes structural alterations through chemical groups such as acetyl, ester, and carboxylic acid (Figure 2b) [72].

For example, the band located at 1600 cm^−1^ attributed to the C=C stretching of aromatic lignin drastically decreases. In this context, the absorption region of the aromatic ring in lignin typically appears in a broader range, between 1600 cm⁻^1^ and 1400 cm⁻^1^, with characteristic peaks indicating the presence of aromatic groups and their substituents. However, depending on the biomass type, lignin can present structural variations that modify the proportions of guaiacyl (G), syringyl (S), and p-hydroxyphenyl (H) units, which shift the FTIR bands in this region. Thus, the peak at 1600 cm^−1^ may contain overlapping signals from the C=C of aromatic rings and the C=O stretching of conjugated carbonyl groups. Consistent with previous works, the intensity of cellulose-related bands indicates that the chemical treatment efficiently removes lignin and hemicellulose components from BPS, as observed in other biomass wastes such as the case of cocoa pod husk [73].

Regarding the structure of cellulose, the hydroxyl groups are involved in a number of intra- and intermolecular hydrogen bonds. These H-Bonds are considered one of the key factors that directly affect the crystallinity index, which, in turn, influences the chemical, physical, and mechanical performance of cellulose for real-world applications. For example, it has been established that the reduction in crystallinity results from the disintegration of intermolecular bonds, causing a blue shift in the carbonyl signal of the FT-IR spectra [74]. For hydrogen bond identification, some techniques are commonly used, such as FT-IR spectroscopy, the in situ FT-IR analysis of the peroxidation process at different temperatures (from room temperature to 350 °C), and molecular simulation. In particular, infrared spectroscopy in the region of 3700 and 3000 cm^−1^ shows overlapping signals corresponding to two types of molecular hydrogen bonds (intra- and intermolecular). For example, peaks close to 3370 cm^−1^ are expected to correspond to intramolecular H-bonds, whereas intermolecular H-bonds typically appear between 3410 and 3400 cm^−1^ and 3600 and 3500 cm^−1^ [66]. Particularly, it has been established that cellulose I exhibits intramolecular H-bonds (O_2_H·O_6_, O_3_H·O_5_) at 3455–3410 cm^−1^ and 3375–3340 cm^−1^, while at 3310–3230 cm^−1^, the intermolecular H-bonds (O_6_H·O_3′_) are displayed, as well as the valence vibrations of N-bonded OH groups at 3570–3450 cm^−1^; these assignments have been corroborated by computational techniques [75] and confirm that the number of H bonds is related to the band intensity of the infrared results, but the infrared band position may vary depending on the source [76]. In the case of cellulose II (with parallel and antiparallel orientations relative to cellulose I), it can exhibit O_6_H·O_2′_ intermolecular H-bonds and O_2_H·O_6_, O_6_H·O_2′_ intramolecular H-bonds (linked to cellulose I) [77,78].

**Figure 3:**
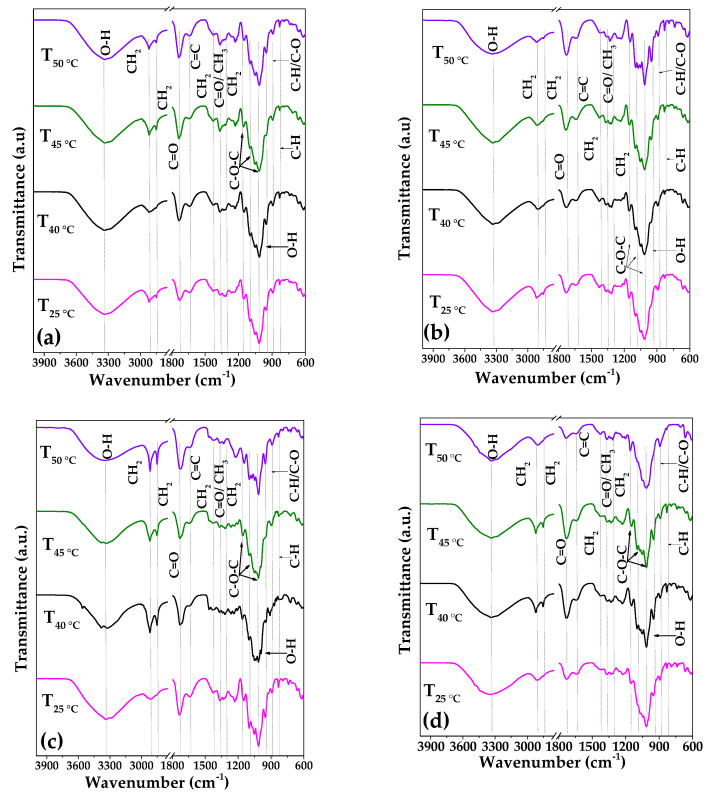
FTIR transmittance spectra for nanocellulose samples pretreated with H_2_SO_4_ at different concentrations of 45 or 55 wt.%, temperatures of 25, 40, 45, or 50 °C, and reaction times of 30 or 60 min. (**a**) CNC-AVB_45wt.%, 30min_, (**b**) CNC-AVB_45wt.%,60min_, (**c**) CNC-AVB_55wt.%,30min_, and (**d**) CNC-AVB_55wt.%,60min_.

**Figure 4:**
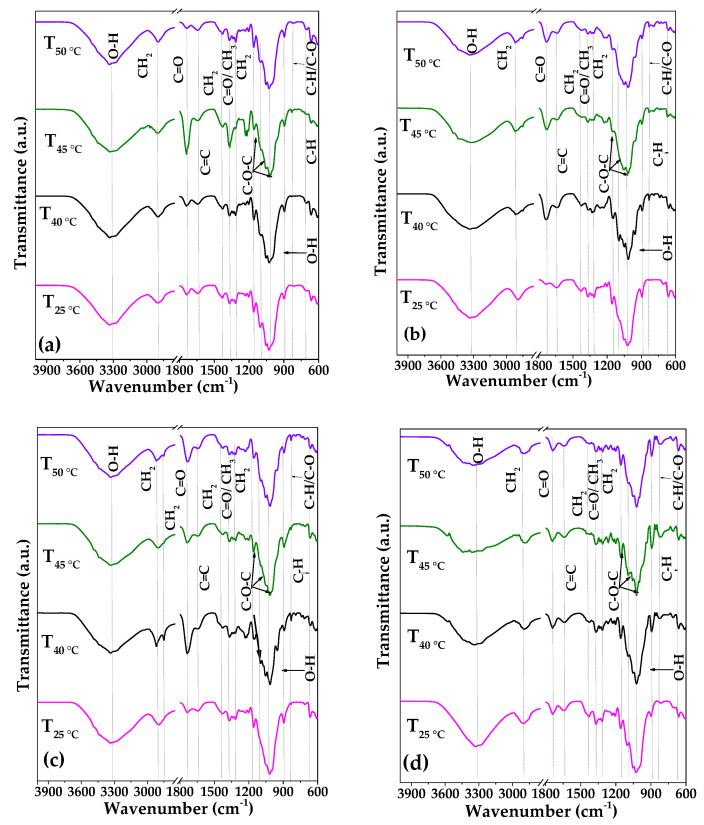
FTIR transmittance spectra for nanocellulose samples pretreated with H_2_SO_4_ at different concentrations of 45 or 55 wt.%, temperatures of 25, 40, 45, or 50 °C, and reaction times of 30 or 60 min. (**a**) CNC-BPS_45wt.%, 30min_, (**b**) CNC-BPS_45wt.%,60min_, (**c**) CNC-BPS_55wt.%,30min_, and (**d**) CNC-BPS_55wt.%,60min_.

Then, it is evident that a specific refinement to assign hydrogen bonds in cellulose is still under debate. For this reason, a systematic analysis was performed in this work, varying acid solution concentration, temperature, and reaction time to evaluate changes in the position of H-bond wavelengths during the process of the isolation of nanocrystalline cellulose. Figure 3a–d and Figure 4a–d show the infrared spectra of nanocrystalline cellulose obtained from AVB and BPS sources under different synthesis conditions; meanwhile, the analysis of the second derivative in the 3600 to 3000 cm^−1^ region, aimed at identifying the types of H-bonds and free OH groups, can be observed in Figures S1 and S2 [66].

**Figure 5:**
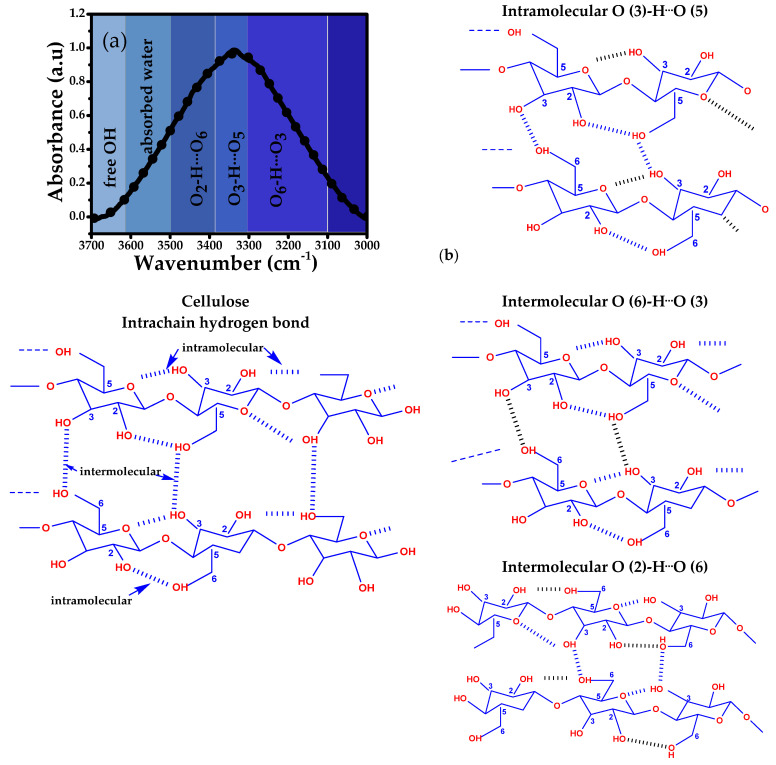
(**a**) FT-IR deconvolution of cellulose from 3000 to 3700 cm^−1^ and (**b**) intramolecular and intermolecular H bonds.

As is well known, a first-order derivative represents the rate of absorption change with respect to wavelength and begins and ends at 0. The second-order derivative corresponds to a negative band with a minimum at the same wavelength as the maximum in the zero-order band; it also displays two additional positive bands on either side of the main band. Assuming that the zero-order spectrum obeys Beer’s law, a similar linear relationship exists between concentration and amplitude for derivative spectra, which are expressed as $\frac{d^{n}A\left(\lambda \right)}{d\lambda^{n}}=\frac{d^{n}\alpha \left(\lambda \right)}{{\left[d\lambda \right]}^{n}}bc$, where *A* is the wavenumber-dependent absorbance, *α* is the absorption coefficient, *b* is the optical path length, and *c* is the sample concentration [63,79]. The second derivative was adjusted in the range of 3600–3000 cm^−1^ using a Gaussian functional model. Figure 5 illustrates the representation of hydrogen bonds (intramolecular and intermolecular) for each cellulose type, and Tables S1 and S2 present the specific fitted data of this analysis.

Table 1 and Table 2 display hydrogen bond energies and distances for intra- and intermolecular bonds based on the results of second derivative analysis.

In general, the as-prepared samples from AVB displayed the O_3_H·O_5_ intramolecular H-bonds in the range from 3312 cm^−1^ to 3394 cm^−1^ (Table 1). Wavenumbers between 3425 and 3511 cm^−1^ correspond to the O_2_H·O_6_ intramolecular hydrogen bonds, and the O_6_H·O_3′_ intermolecular H-bonds were located between 3115 and 3299 cm^−1^. Similarly, the range of the samples from BPS was 3312–3339 cm^−1^ (O_3_H·O_5_), 3327–3493 cm^−1^ (O_2_H·O_6_), and 3138–3309 cm^−1^ (O_6_H·O_3′_) for intramolecular and intermolecular hydrogen bonds, respectively (Table 2).

As expected, both energies and distances of hydrogen bonds vary slightly with the type of source and experimental conditions due to the beating degree, which can modify the final crystallinity index. For example, variation in H-bond energies of the O_6_H·O_3_ bond can reach up to 33.60% depending on the experimental conditions: from 25.53 to 38.47 kJ mol^−1^ in the case of AVB and from 25.16 to 37.39 kJ mol^−1^ for BPS. Furthermore, the H-bond distances in the different bond types and free OH groups show variations, demonstrating that, under experimental conditions, cellulose can exhibit different properties. In particular, the distances of hydrogen bonds ranged between 2.73 and 2.83 Å for AVB and between 2.73 and 2.83 Å for BPS, representing variations of 3.70 and 3.40%, respectively. The results suggest that both sources predominantly contain crystalline cellulose type I [63,80].

The variations in the free O_2_H and O_6_H groups were correlated with the quantity of adsorbed water, which is modified by the experimental parameters used during the isolation process. The occurrence of intermolecular hydrogen bonds in the samples is expected to enhance the strength of the bonds between cellulose fibers, thus improving mechanical properties, while intramolecular hydrogen bonds reinforce the cohesion of cellulose molecules, facilitating the penetration of chemical reagents and improving the crystalline areas. When analyzing the relative content of the three types of hydrogen bonds and free OH groups in AVB and BPS, it is observed that, in general, both sources display higher proportions of O_3_H·O_5_ and O_2_H·O_6_ intramolecular bonds, although the O_6_H·O_3′_ intermolecular bonds increase significantly when using BPS as the source (Supplementary Materials, Tables S3 and S4). The analysis confirms that, although both types of H-bonds exist, the crystallinity index is mainly determined by intramolecular hydrogen bonds. It is likely that, during the experimental procedure, O_6_H·O_3′_ bonds are broken to form new H-bonds, even in amorphous areas [66].

### 3.2. Yield of Nanocellulose

#### 3.2.1. Effect of Reaction Time

The cellulose obtained was subjected to acid hydrolysis using H_2_SO_4_ at different concentrations, reaction times, and temperatures to regenerate nanocellulose. In this sense, the yield of nanocellulose from aloe vera bagasse and banana pseudostem was determined for its application in the manufacture of hydrogels. As can be seen in Table 3, the nanocellulose yield increases as hydrolysis time decreases. Specifically, the sample AVB C_45_t_30_T_25_ achieved a yield of 47.63% ± 1.29 after 30 min of reaction; at 60 min, the yield slightly increased to 49.65% ± 5.21. Similarly, the BPS sample C_55_t_30_T_25_ showed a yield of 54.31% ± 2.40 after 30 min, while the sample C_55_t_60_T_25_ (BPS) showed yield values around 55.18% ± 0.03. This behavior might be attributed to the fact that longer times cause the chemical agents to disintegrate the amorphous regions more extensively.

#### 3.2.2. Effect of Acid Concentration

The variation of acid concentration did not show an important effect on the cellulose yield. For example, when a concentration of 45% *w*/*w* was used, the isolated cellulose from AVB was found, in the best case, to be 49.90 ± 5.86% (C_45_t_30_T_45_), whereas, using a concentration of 55% *w*/*w*, the yield was quite similar, reaching 48.85 ± 0.92% (C_55_t_30_T_45_). Similarly, using BPS as a source showed the highest yields under the C_45_t_30_T_45_ conditions, with 59.21 ± 2.50%, and this value was slightly reduced with an increase in acid concentration (55% *w*/*w*) under similar experimental conditions 58.26 ± 2.09% (C_55_t_30_T_45_). The small variations of the cellulose yield with the acid concentration have previously been related to the dehydrating action on cellulose and/or the inability to rupture glycosidic linkages [81]. In fact, at this point, it is clear that the source conditioned the final performance of the biopolymer.

#### 3.2.3. Effect of Temperature

Regarding the effect of temperature, some researchers have reported that the crystalline regions can degrade when the temperature increases, resulting in lower yields [82]. In the evaluated samples, this phenomenon was observed in some AVB samples; for example, maintaining the concentration at 55% *w*/*w* and a reaction time of 60 min, the yields as the temperature increased were 49.61% ± 4.79 (25 °C), 45.06 ± 9.01 (40 °C), 46.19 ± 0.92 (45 °C), and 46.65% ± 1.30 (50 °C). For the AVB source, the optimal conditions to obtain a maximum yield were achieved at C_45_t_30_T_45_ (49.90% ± 5.86). In the case of BPS, the maximum yield was observed at C_45_t_30_T_45_ (59.21% ± 2.50). In summary, a combination of factors contributes to achieving better yields during the isolation of cellulose, but it also depends on the source material. Interestingly, the yield reported in this study is relatively higher than other works reported by various researchers [83,84,85]. Table S5 shows a comparison of nanocellulose yields obtained from diverse biomass sources under different extraction conditions; for instance, Sartika et al. reported a corn cob cellulose yield of 50.07%, utilizing acid hydrolysis with 30 wt.% of H_2_SO, at a temperature of 50 °C, and a reaction time of 30 min [86,87]. Likewise, low yields were observed in the work reported by Coelho et al., who obtained a cellulose yield from grape pomace of 12% [88]. In general, these results highlight the necessity of understanding the influence of the various factors, such as biomass type (cellulose content, hemicellulose and lignin) and extraction conditions (concentration, time, and temperature), to achieve significant yields in obtaining nanocellulose.

### 3.3. Dynamic Light Scattering (DLS)

**Figure 6:**
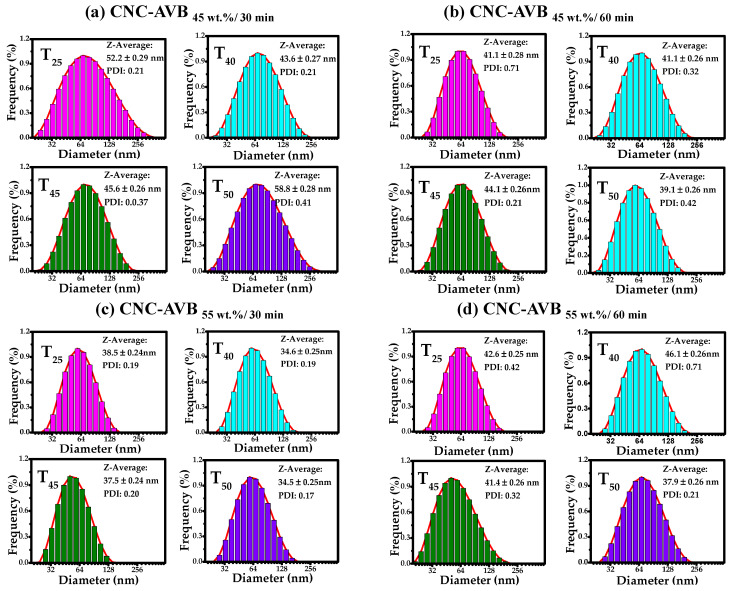
Hydrodynamic diameter distribution histograms of CNC obtained at different acid concentrations, reaction times, and temperatures of 25, 40, 45, and 50 °C. (**a**) CNC-AVB_45wt.%, 30min_, (**b**) CNC-AVB_45wt.%, 60min_, (**c**) CNC-AVB_55wt.%, 30min_, and (**d**) CNC-AVB_55wt.%, 60min_.

**Figure 7:**
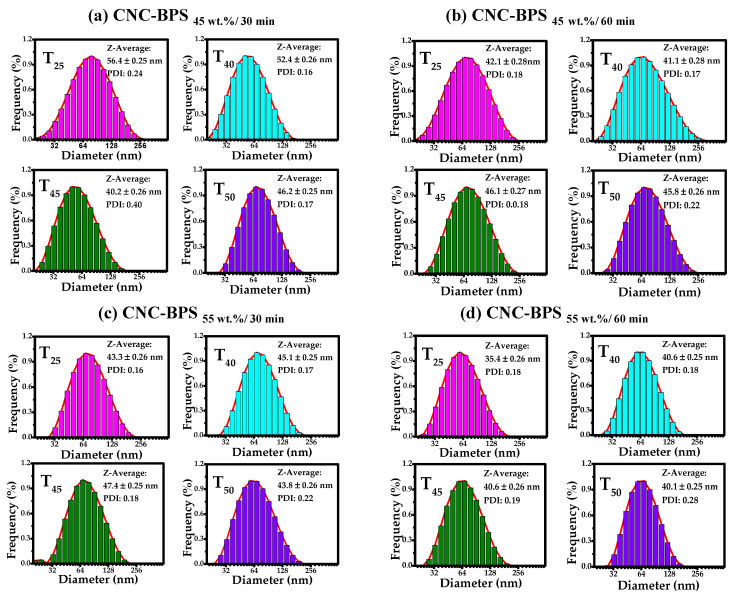
Hydrodynamic diameter distribution histograms of CNC obtained at different acid concentrations, reaction times, and temperatures of 25, 40, 45, and 50 °C. (**a**) CNC-BPS_45wt.%, 30min_, (**b**) CNC-BPS_45wt.%, 60min_, (**c**) CNC-BPS_55wt.%, 30min_, and (**d**) CNC-BPS_55wt.%, 60min_.

The relative hydrodynamic diameter distribution of the CNC was analyzed by dynamic light scattering (DLS). It is well known that nanocellulose easily agglomerates when dispersed in an aqueous solution; thus, it is crucial to determine ultrasonication time and dilutions to evaluate stable solutions [89]. After analyzing different sonication times, the most stable solution was obtained after 60 min of ultrasonication [90,91]. Additionally, different dilutions were evaluated, and a high dispersion was obtained with a dilution of 10^−13^. Nanocellulose from both sources (AVB and BPS) isolated under different conditions was analyzed in water using DLS (Figure 6a–d and Figure 7a–d).

When comparing both sources for obtaining CNC, it was observed that AVB specimens show a tendency to decrease the hydrodynamic diameter compared to BPS. For example, the low hydrodynamic diameter sizes were found to be about 38.50, 34.60, 37.50, and 34.50 nm for the samples C_55_t_30_T_25_, C_55_t_30_T_40_, C_55_t_30_T_45_, and C_55_t_30_T_50_, respectively, while the highest hydrodynamic diameter was observed with CNC-AVB_45wt.%, 30min_ at 52.20 (25 °C), 43.60 (40 °C), 45.60 (45 °C), and 58.80 nm (50 °C). On the other hand, the hydrodynamic diameter for isolating CNC from BPS was obtained, at best, with CNC-AVB_55wt.%, 60min_, 35.4 (25 °C), 40.60 (40 °C), 40.60 (45 °C), and 40.1 nm (50 °C), and the largest size was obtained with the samples CNC-BPS_45wt.%, 30min_ with hydrodynamic diameters of 56.40, 52.40, 40.20, and 46.20 nm when temperatures of 25, 40, 45, or 50 °C were used during the acid treatment.

DLS analyses indicate that the average size of cellulose nanoparticles varies between 30 and 60 nm, although the histograms show bars that present some particles with diameter sizes between 10 and 200 nm. Considering the size of the hydrodynamic radio, the interval confirms that nanocellulose was isolated successfully [92]. Furthermore, compared with other sources, the results demonstrate that the average values of hydrodynamic diameter are lower than those of banana (85–125 nm) [93] and the biomass of cocoa pod husk (from 41 to 155 nm) [73]. Regarding the polydispersity index (*PDI*), the obtained nanocellulose exhibited *PDI* values from 0.20 to 0.71. Although few samples reached the maximum value, this suggests a low overall polydispersity [94]. As is known, values close to 0 refer to monodisperse, while 1 is assigned to polydisperse. *PDI* values are comparable to those obtained in the isolation of cellulose from Butia fruit reported previously (*PDI* = 0.20); however, the specimens display a homogeneous particle size distribution [95].

### 3.4. X-Ray Diffraction

**Figure 8:**
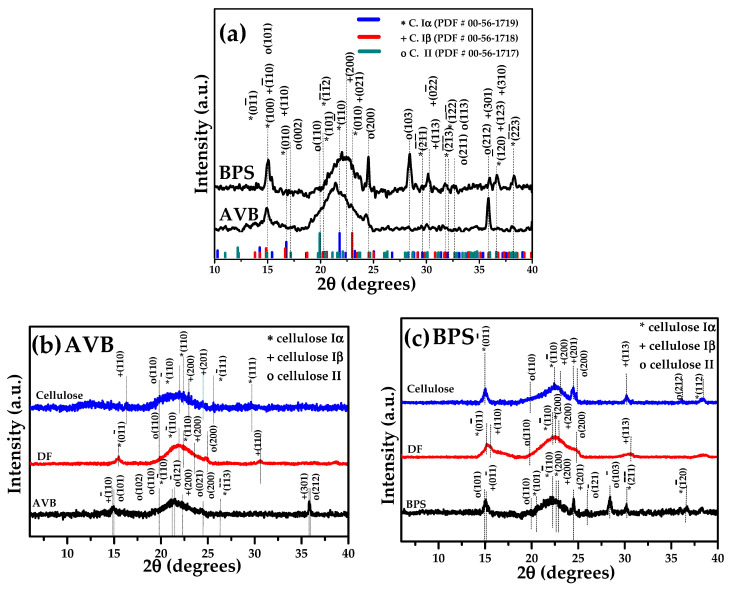
X-ray diffractograms of (**a**) raw materials, AVB (aloe vera bagasse), and BPS (banana pseudostem) and its comparison with the untreated fibers (UF), delignified fibers, and cellulose from (**b**) AVB and (**c**) BPS.

Figure 8 shows XRD patterns during the CNC processing of BPS or AVB agro-industrial waste. The figure shows XRD spectra for samples without treatment (WT), during the delignification of fibers (DF) and cellulose fibers, and, finally, the isolation of CNC. For the analysis of the polymorph phases of cellulose, the crystallographic charts PDF#00-561719, 00-561718, and 00-561717 were used. These charts correspond to the triclinic phase of cellulose I alpha as well as the monoclinic phase of I beta and cellulose II, respectively.

Untreated AVB and BPS fibers show peaks corresponding to three cellulose polymorphs; the observed cellulose signals were I_α_ at θ-2θ of 14.77° $\left(110\right)$, 26.32° $\left(1\;\bar{1}\bar{3}\right)$. For cellulose I_β_, the peaks can be observed at 22.39° $\left(200\right)$, 35.80° $\left(301\right)$, and, finally, the reflections for cellulose II were identified at 14.97°, 21.19°, 21.48°, 23.44°, 24.50°, and 35.90° according to $\left(101\right)$, $\left(102\right)$, $\left(1\bar{2}1\right)$, $\left(021\right)$, $\left(200\right)$, $\left(212\right)$ planes, respectively. As can be seen, BPS fibers present signals with higher intensity compared to AVB fibers; for example, the signals (−110), (200), (110) correspond to the phases I_α_ (21.80°), I_β_ (22.98°), and II (19.92°), respectively. The differences in intensity are correlated with the intrinsic properties of the biomass used.

After the delignification process, some signals of XRD patterns are missing, indicating that the elimination of lignin and hemicellulose during the chemical treatment was carried out effectively. The crystallinity of the cellulose reflections was analyzed by deconvoluting the signals in the *θ*–2*θ* range of 10–30° using the Gaussian method (Figures S3 and S4). The characteristic diffraction signals corresponding to the types of cellulose under the different experimental conditions to obtain CNC, as well as crystallite size (*C.S.*) and crystallinity index (*CI*), are shown in Table 4. From these data, all the experimental conditions for BPS samples displayed signals that matched well with the I_α_ and I_β_ phases, which can be observed at 15.42°$\left(0\bar{1}1\right)$, 18.65°$\left(\bar{1}\bar{1}1\right)$, 20.22°$\left(\bar{1}\bar{1}2\right)$, and 21.80°$\left(\bar{1}10\right)$, and 13.80°(011), 14.27°(101), 14.85°$\left(\bar{1}10\right)$, 16.66°(110), and 22.98°(200), respectively. The intensity of these signals varies depending on the synthesis conditions but still preserves the crystallinity of cellulose II $\left(100\right)$ at ~12.26°. The same goes for the CNC obtained from the AVB source, where the I_α_ phase appears at 14.26°$\left(100\right)$, 15.24°$\left(0\bar{1}1\right)$, 18.65°$\left(\bar{1}\bar{1}0\right),$ 20.63°$\left(002\right)$, and 21.80°$(\bar{1}10$) and the cellulose I_β_ at 13.80°$\left(011\right)$, 14.27°(101), 14.85°$\left(\bar{1}10\right)$, 16.66°$\left(110\right)$, 18.74°(111), 20.27°$\left(012\right)$, and 22.98°(200). In the case of cellulose II, a greater number of signals can be observed, 12.26°(100), 14.94°(101), 17.18°(002), 19.92°(110), 19.77°$\left(\bar{1}20\right),\;21.13{}^{\circ}\left(102\right)$, and 21.58°$\left(\bar{1}21\right)$, which highlights the differences in the composition of each biomass to obtain CNC.

The deconvolution of the signals provides a more reliable estimate of crystallinity; the signals were fitted to Gaussian functions, and the crystallinity index was calculated using the Segal equation (Table 4, see also deconvolution figures in the Supplementary Materials, Figures S3 and S4). The CNC from BPS showed *CI* % from 72.47 to 97.58% under the evaluated conditions. These values are higher than those reported by Mishra et al., whose crystallinity values were reported to be about 62.18% using banana peel extraction [96], or those reported by Merais et al., with values of 65.60 and 75.37% for other types of banana species: *M. acuminata* and *M. balbisiana* [97].

CNC synthesized from AVB showed a crystallinity percentage from 70.67 to 89.66%. Nanocrystalline cellulose from banana pseudostem showed higher crystallinity values compared to CNC from aloe vera bagasse, which can be correlated with the composition of each source [98]. As expected, this combination of biomass to obtain CNC and the synthesis conditions can modulate the final crystallinity percentage. For example, CNC from rice husk showed 72% crystallinity when chemical acid hydrolysis was combined with pressure homogenization, but was reduced at atmospheric pressure [99], while the CNC from acai bagasse (*Euterpe oleracea*) presented a crystallinity percentage of 62% when different chemical reagent ratios (H_2_SO_4_/HCl) were mixed during acid hydrolysis [100]. On the other hand, the reaction time is an important factor in hydrolysis treatments: a shorter time can result in the partial elimination of amorphous regions and a longer time causes a lower yield and the better removal of the amorphous regions [101]. During chemical treatment with sulfuric acid (H_2_SO_4_), it reacts with the fiber’s amorphous region, causing the hydrolytic rupture of the glycosidic bonds and the release of individual crystals, which, in turn, causes the growth of single crystals that contribute to the increase in crystallinity [102,103]. It is clear that it is necessary to control the acid solution concentration and temperature of reaction to obtain a maximum crystallinity percentage. For example, CNC from BPS at C_45_t_30_T_50_ displayed crystallinity of 87.76%, and this increased to about 97.58% for C_55_t_30_T_50_. Temperature did not show a clear trend in the crystallinity. Table 4 also presents the crystal size calculated by the Scherrer equation. For the CNC-BPS sample, the size obtained is in the range from 23.40 to 68.30 nm and, for the CNC-AVB sample, from 27.20 to 39.10 nm. The results confirm that the isolation of CNC from these sources is efficient when synthesis conditions are optimized. In this case, the maximum crystallinity in all the samples reached up to 97.58% with the conditions of C_55_t_30_T_50_.

### 3.5. Scanning Electron Microscope (SEM)

**Figure 9:**
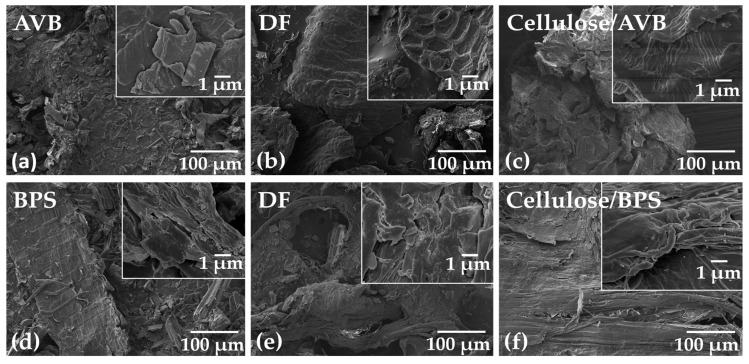
Selected SEM images of the samples after each step to obtain cellulose fibers (**a**) aloe vera, (**b**) delignified fibers from aloe vera, (**c**) cellulose from aloe vera, (**d**) banana pseudostem, (**e**) delignified fibers from banana pseudostem, and (**f**) cellulose from banana pseudostem.

The changes in the morphology of specimens after each treatment step were observed using SEM (Figure 9a–f). The biomass fibers exhibited a smooth surface and compact structure; specifically, in the AVB sample, cells belonging to the epidermis and parenchyma can be observed (Figure 9a). Brick-shaped cells that are part of the epidermis that make up the exocarp can also be appreciated [104,105,106]. After treatment, the aloe vera fiber presents a separation and development of porosity (Figure 9b,c). This behavior is related to the removal of wax, lignin, and hemicellulose components [107]. Selected BPS micrographs are shown in Figure 9d–f. Tissues of the vascular bundle, as well characteristic epidermal cells and cell complexes that constitute the vascular and supporting tissue, were identified [104]. Additionally, the fibers are relatively smooth and orderly. Their surface is covered with wax, pectin, hemicellulose, and lignin, which constantly interact with the cellulose (Figure 9d) [108]. In addition, some fibers present roughness and fragmentation due to the size reduction applied before chemical treatment (Figure 9e). It can be seen that the surface of the sample was fragmented, became rough, and holes began to appear; these changes indicated the elimination of hemicellulose and lignin bonds, which caused superficial cracks, affecting the internal layers. Cellulose fibers displayed a morphology with greater cracking and surface fragmentation compared to DF; this is generated during the isolation of cellulose fibers, particularly due to the reaction of the O-H groups with lignin (Figure 9f) [108]. After the delignification process, various components (pectin, waxes, extractives, polyphenols, hemicellulose, and other polysaccharides) were removed; therefore, the surface of the fiber is more exposed to acid hydrolysis.

**Figure 10:**
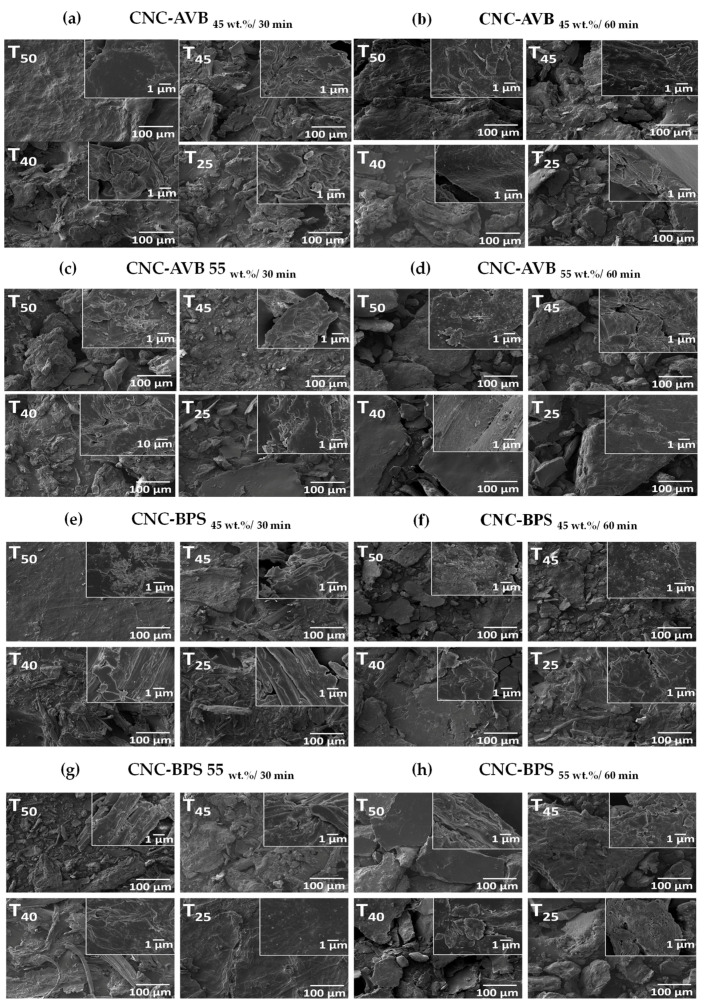
Selected SEM images CNC synthesized at different temperatures (25, 40, 45, or 50 °C), acid solution concentrations (45 and 55 wt.%), and reaction times (30 and 60 min). (**a**) CNC-AVB_45wt.% 30_, (**b**) CNC-AVB_45wt.%-60min_, (**c**) CNC-AVB_55wt.%-30min_, (**d**) CNC-AVB_55wt.%-60min_, (**e**) CNC-BPS_45wt.%-30min_, (**f**) CNC-BPS_45wt.%- 60min_, (**g**) CNC-BPS_55wt.%-30min_, and (**h**) CNC-BPS_55wt.%- 60min_.

Regarding the acid hydrolysis treatment (sulfuric acid H_2_SO_4_ of 45 wt.% or 55 wt.%, temperatures of 25, 40, 45, or 50 °C, and reaction times of 30 or 60 min), an analysis of the morphological changes on the CNC was carried out depending on the synthesis conditions (Figure 10a–h). It can be seen that the micrographs of the CNC samples that present the highest fragmentation were from the CNC-BPS _55wt.%, 50 °C, 30min_ sample, which also presents fiber agglomeration in the form of flakes due to strong hydrogen bonds in the fibers during the drying process [109]. According to previous studies, it seems that temperature modified the morphological appearance; the increase in temperature generates a partial defibrillation, and the opening of fiber bundles, as can be seen for the CNC-BPS samples [110]. In this context, the defibrillation causes the degradation of glycoside and ether bonds in cellulose, eliminating some amorphous regions during the interaction with hydronium ions (H^+^) that are released during acid hydrolysis. Thus, cellulose fibers fragment into fine fibrils, i.e., the elimination of these amorphous sections allowed us to obtain a more crystalline material. In general, the obtained results confirm that the applied conditions were efficient in causing changes in the surface of the material for the isolation of cellulose from both types of biomass. The morphological studies agreed with other reported works [108], where the removal of hemicellulose, pectin, and lignin provoked important changes in the morphological aspects of CNC.

### 3.6. FT-IR Analysis of Hydrogels

**Figure 11:**
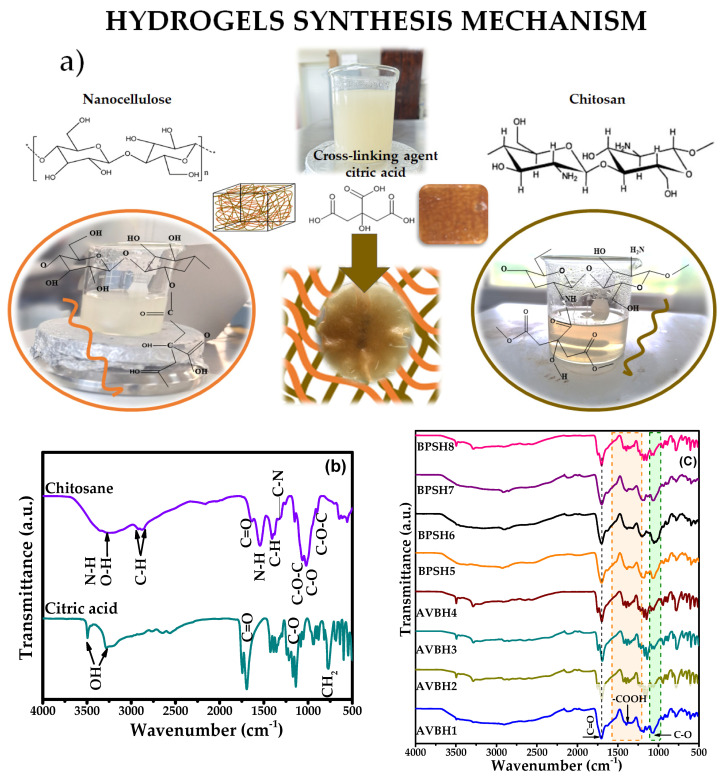
(**a**) Representation of the synthesis of hydrogels, (**b**) FTIR spectra of chitosan and citric acid, and (**c**) FTIR spectra of hydrogels obtained from AVB: H1 (C_45_t_30_T_50_), H2 (C_45_t_60_T_40_), H3 (C_55_t_30_T_50_), and H4 (C_55_t_60_T_45_) and, for the hydrogels, from BPS H5 (C_45_t_30_T_50_), H6 (C_45_t_60_T_40_), H7_5_(C_55_t_30_T_2_), and H8_0_ (C_55_t_60_T_5_).

The hydrogels were synthesized by the methodology proposed above and illustrated in Figure 11a. The used nanocellulose to produce hydrogels was chosen based on the percentage of crystallinity; thus, the samples with high *CI* %, in the case of AVB as a source, were C_45_t_30_T_50_ (H1), C_45_t_60_T_40_ (H2), C_55_t_30_T_50_ (H3), and C_55_t_60_T_45_ (H4) and, for the nanocellulose from BPS, the selected samples were _C45_t_30_T_50_ (H5), C_45_t_60_T_40_ (H6), C_55_t_30_T_25_ (H7), and _C55_t_60_T_50_ (H8).

The Fourier transform infrared spectroscopy (FT-IR) of the hydrogel and the agents used as a base for its synthesis are shown in Figure 11b,c. In the case of chitosan, FT-IR spectra show characteristic N-H functional groups, as well as the stretching vibration of the O-H group and intramolecular hydrogen bonds, which are overlapped between 3377 cm^−1^ and 2987 cm^−1^. In the region between 2972 and 2827 cm^−1^, the symmetric and asymmetric stretching of the C-H functional group appears. The signal located at 1640 cm^−1^ corresponds to the stretching vibration of C=O groups contained in amide I, whereas the absorption band corresponding to the N-H bending from amide II is located at 1548 cm^−1^ [111]. The C-H deformation is also observed at 1417 cm^−1^. Similarly, at 1310 cm^−1^, the absorption band of the C-N functional group stretching of amide III appears, confirming the presence of residual N-acetyl groups [112,113]. The signals located at 1061cm^−1^ represent the stretching of the C-O-C bridge vibration; likewise, the absorption signal located at 1005 cm^−1^ is attributed to the stretching vibrations of the C-O groups [114]. Finally, the C-O-C functional groups located at 885 cm^−1^ are attributed to chitin.

On the other hand, for citric acid (Figure 11b), the wide band between 3500 and 3200 cm^−1^ corresponds to the stretching of O-H, and the signals at 1740 cm^−1^ and 1690 cm^−1^ correlate with the C=O stretching vibrations [115]. The wavenumber range of between 1267 and 1015 cm^−1^ is correlated with C-O stretching, and CH_2_ rocking appears at 770 cm^−1^.

By evaluating the changes in the bands of the carboxylic groups, the linkages between nanocellulose and citric acid can be confirmed, which is essential in the performance of hydrogels. Figure 11c shows the spectra of the synthesized hydrogels; possible changes in the intensity of functional groups and their interaction with the cross-linking agent during the synthesis are highlighted. The interaction between CNC, chitosan, and citric acid in the hydrogel synthesis was confirmed by the FTIR analysis through some additional or decreased signals. For example, during the esterification reaction of citric acid with cellulose and chitosan, the consumption of O-H functional groups occurs, which is observed as a drastic decrease in the absorption band between 3700 cm^−1^ and 3000 cm^−1^ [116,117].

Also, the bands at 2916 cm^−1^ and 2844 cm^−1^ that correspond to the symmetric and asymmetric stretching of cellulose and chitosan tend to disappear, suggesting possible interactions involving N-H groups. Furthermore, the band centered at 1737 cm^−1^ indicates the presence of a signal derived from the stretching of functional groups coming from the carboxyl group of citric acid [118]. As can be seen, the hydroxyl and carboxyl groups are the key functionalities supporting adequate hydrogel formation. Additionally, the observed bands from around 1580 cm^−1^ to 1200 cm^−1^ confirm the cross-linking of chitosan and citric acid through carboxyl groups (-COOH). Likewise, the characteristic band located around 1000 cm^−1^ corresponds to C-O stretching groups. The presence of this band confirms the esterification reaction between nanocellulose and citric acid through the interaction of carboxyl groups (-COOH) and hydroxyl groups (-OH) [111].

### 3.7. Swelling, Gel Percentage, and Porosity of Hydrogels

The gel yield obtained for the hydrogels was determined as a function of the extraction properties of cellulose. Considering that this value represents the efficiency of the gelation process, a high gel yield indicates that materials have been successfully incorporated into the hydrogel network. All synthesized hydrogels displayed yields above 80%, indicating adequate gel yield for diverse applications. Particularly, in the case of AVB, the samples displayed a decreasing yield in the following order: H1 (85.78 ± 3.80) > H3 (85.24 ± 2.71) > H2 (84.18 ± 2.51) > H4 (81.87 ± 3.30). Conversely, in the case of BPS, the samples with high yield were H6 (87.28 ± 2.06), H5 (86.36 ± 3.89), H8 (86.65 ± 3.81), and H7 (85.62 ± 3.90).

**Figure 12:**
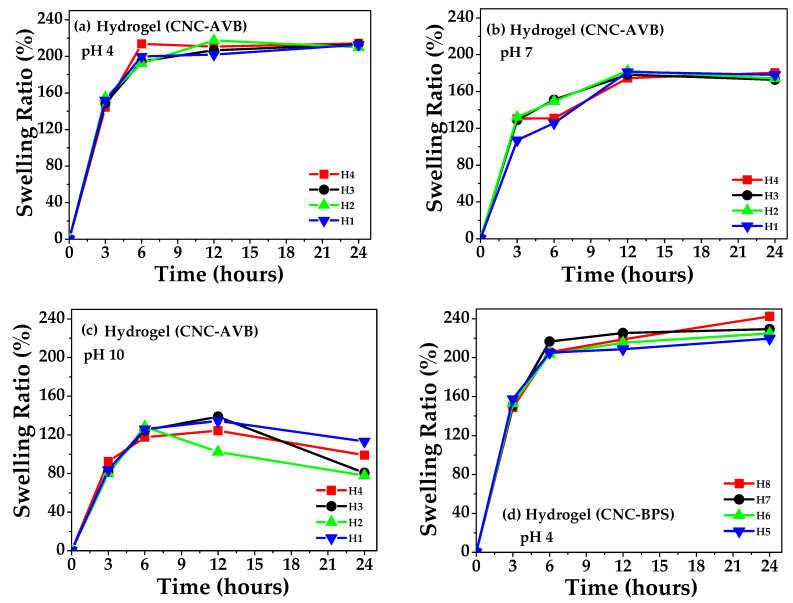

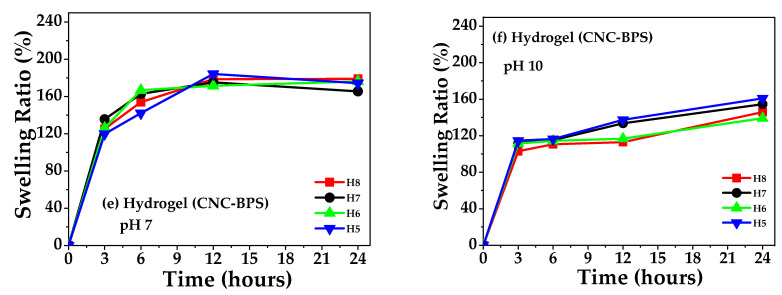
Swelling test of hydrogels: (**a**–**c**) hydrogel CNC- AVB (*pH* 4, 7, and 10) and (**d**–**f**) hydrogel CNC-AVB (*pH* 4, 7, and 10).

The water absorption capacity of the hydrogels was determined to evaluate their swelling behavior in buffered saline. The obtained results are shown in Figure 12a–f. Initially, at 6 h, the hydrogels showed relatively rapid swelling until reaching a certain equilibrium in all the samples after 12 h at pH 4. This behavior was correlated with the interaction of N-H and O-H functional groups. Furthermore, the adsorption capacity increases due to the surface area of the CNC itself [111]. Increasing the *pH* to 7 and 10 slightly modifies this equilibrium after 12 h owing to chitosan acidification, showing swelling variations. That is, an increase in the number of protons in the medium reduces the affinity for water. Previously, it was found that, at *pH* > 6.5 the amino groups of chitosan are deprotonated, which decreases the solubility of the polymer [119], while Peng et al. suggested that the high water retention property can be attributed to the hydrophilic nature of chitosan [120] combined with the chemical composition of the hydrogel. Thus, these features condition the cross-linking density and hydrogel mesh size [121]. The values obtained in the present study showed good absorption capacity, indicating that the cross-linking between citric acid generated the availability of a greater volume for the diffusion of molecules, confirming the *pH* sensitivity of the as-prepared hydrogels.

Therefore, it is assumed that the water absorption is mainly attributed to the hydrophilic groups present in the hydrogel, as well as to the electrostatic repulsion of the components. For hydrogel applications, this property is favorable in wound healing because it maintains moisture and prevents the formation of dry scabs and pain during dressing changes [122,123].

A specific analysis of the as-obtained samples indicates a decreasing order of hydrogel swelling (Table 5). From the AVB source and pH 4, the order found was H2 > H4 > H3 > H1, whereas, for hydrogels from BPS, the following order was observed: H7 > H8 > H6 > H5. Supplementary Information for the samples can be found in Table S6, where the obtained data of this study were compared with other research in the literature. For example, swelling has been reported to be *pH*-dependent for other materials such as starch-polyacrylate-based hydrogel. In this case, it was found that the carboxylate groups are responsible for increasing the adsorption capacity at high *pH* values; the carboxylate groups ionize, causing electrostatic repulsion between them, which, in turn, provokes an increase in swelling in the hydrogel [124]. On the contrary, Carvalho et al. produced hydrogel from bacterial cellulose, suggesting that the swelling capacity of the specimens is strongly influenced by the surface area and porosity, rather than the chemical groups [125], while Ramírez Carmona et al. reported that hydrogel thickness is another important factor that conditions the swelling of the hydrogel. In general, the authors found that hydrogel swelling increases as the film thickness decreases, which can vary from 68% to 93% when used with thicknesses between 0.50 and 0.33 mm, respectively [126].

It is clear that, under the experimental conditions explored in this study, a performance superior to the aforementioned works was found, suggesting that the production of CNC hydrogels from these sources creates a material that can be used for exudative wounds. The gel fraction test was computed to estimate the degree of cross-linking between components and to determine the insoluble part of the hydrogel. From Table 5, it was observed that the hydrogel labeled as H8 (C_55_t_60_T_50_) showed the highest gel fraction (86.60 ± 2.62%) followed by the sample H6 (C_45_t_60_T_40_) 85.94 ± 1.86%. On the contrary, the sample that showed the lowest percentage of gel was H4 (C_55_t_60_T_45_) 77.93 ± 1.88%. These results indicated that the hydrogels with nanocellulose from banana pseudostem have strong chemical cross-linking compared to hydrogel obtained from aloe vera nanocellulose. The results coincide with other research that concludes that the high gel fraction values are due to a higher frequency of junction points between the binder during gelation and ensure good chemical cross-linking in nanocellulose systems reinforced with other components such as chitosan, starch, and wood ash, even with soil applications [111,127].

### 3.8. Morphology of Hydrogels

**Figure 13:**
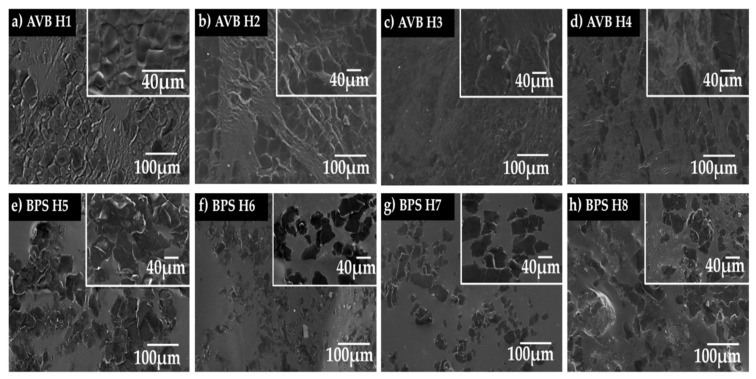
SEM images of hydrogels: (**a**) hydrogels of nanocellulose from aloe vera bagasse CNC-AVB C_45_t_30_T_50_, (**b**) hydrogel CNC-AVB C_45_t_60_T_40_ (H2), (**c**) hydrogel CNC-AVB C_55_t_30_T_50_ (H3), (**d**) hydrogel CNC-AVB C_55_t_60_T_45_ (H4), and, for the hydrogels of nanocellulose from banana pseudostem, CNC- BPS (**e**) hydrogel CNC-BPS C_45_t_30_T_50_ (H5), (**f**) hydrogel NC-BPS C_45_t_60_T_40_ (H6), (**g**) hydrogel NC-BPS C_55_t_30_T_25_ (H7), and (**h**) hydrogel CNC-BPS C_55_t_60_T_50_ (H8).

The morphological features of the hydrogels are shown in Figure 13a–h; these materials presented a compact or dense surface in some areas, which can be correlated to an increased cross-linking in the hydrogel network. The compact and dense surface provides rigidity, so the structure can improve the mechanical properties of the hydrogel [128]. In the remaining areas, the hydrogel exhibited a wrinkled surface and pores of different sizes correlated with the water retention capacity.

Porosity is the main characteristic of hydrogels for maintaining their structural properties; the interconnection between pores and their size plays a crucial role in facilitating the diffusion processes of agents. The porosity of the studied hydrogel varied between 50 and 60%; as can be seen in Table 5, there are changes in porosity depending on the synthesis conditions. The highest porosity values were observed for the CNC-BPS hydrogels at 58.20 ± 1.46%, 60.77 ± 2.60%, 58.80 ± 0.97%, and 57.37 ± 0.86% for the samples H5, H6, H7, and H8, respectively. Meanwhile, for CNC-AVB hydrogels, the obtained values were H1 53.45 ± 1.24%, H2 55.09 ± 0.90%, H3 54.89 ± 0.67%, and H4 53.37 ± 6.28%. These results are consistent with other novel dressing materials manufactured in recent years, such as the poloxamer hydrogel used for the re-epithelization of skin injuries, which showed porosity values between 36.47 ± 0.74% and 41.44 ± 4.19% [69]. However, the porosity values were lower compared to other reported data (Table S6). The mismatch can be attributed to two factors, the crystallinity of the samples and the high cross-linking observed, which results in a less porous surface. Recent studies claim that the pore size decreases with increasing concentrations of nanocellulose crystals, which generates a change in the porosity value attributed to physical cross-linking as well as the CNC structure. However, with an increase in CNC concentration, the network structure within the hydrogel becomes denser, resulting in the higher stiffness of the polymer chains [129]. The average pore sizes for CNC-AVB hydrogels ranged from 2.26 to 3.15 μm and from 2.45 to 2.81 μm for CNC-BPS hydrogels. According to the IUPAC, these pore sizes (*ø*) are classified as macropores because they have a size greater than 50 nm [130]. Similar results were reported by Muhammad Rizwan; the hydrogel synthesized from *Acer platanoides* cellulose exhibited pores sizes ranging from 2 to 25 μm. The authors found that a porous surface favors greater absorption capability and, consequently, a greater swelling ratio [131]. Generally, the porous structures of hydrogels in the range of 3–20 μm play a crucial role in enhancing the proliferation of epidermal keratinocytes and fibroblast cells during healing due to the distribution of different soluble nutrients in the culture medium or wound substrate between the cells. Although pore sizes within the range of 20–125 μm can ensure water permeability and help skin regeneration by providing oxygen and nutrients necessary for cell adaptation [62], they can also increase the risk of bacterial contamination [132]. Thus, the obtained results fall within the appropriate range for the application of hydrogels for wound healing.

### 3.9. Thermal Analysis of Hydrogels

**Figure 14:**
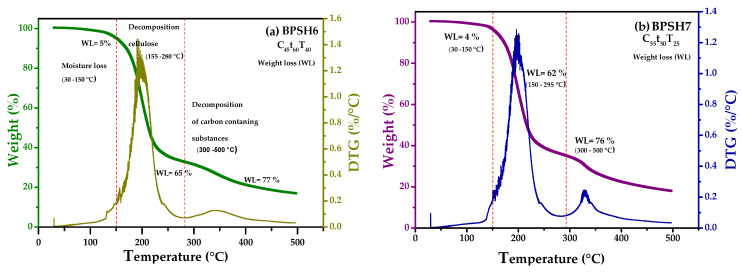
Thermogravimetric analysis (TGA) and derivative thermogravimetry (DTG) of selected samples (**a**) C_45_t_60_T_40_ (H6) and (**b**) C_55_t_30_T_25_ (H7), where the weight loss as well as derivative of change in mass as a function of the temperature can be observed.

The thermal degradation of hydrogels was analyzed by thermogravimetric analysis (TGA) in a temperature range from 30 to 500 °C. Thermal stability properties of hydrogels were discussed in terms of the mass relationships of components during the different degradation stages. The TGA and derivative thermogravimetry (DTG) curves showed three main weight loss stages (Figure 14a,b).

The first weight loss (*WL*), located from 30 to 150 °C, corresponds to a ~5% evaporation of internal water in all hydrogels. The second degradation stage, from 155 to 280 °C, with a greater weight loss indicated by DTG at 190 °C, was attributed to the thermal degradation of nanocellulose, showing significant mass losses of ~65% and ~62% for hydrogel H6, composed of 45% banana pseudostem nanocellulose, and H5, with 55% banana pseudostem nanocellulose, respectively. These results are consistent with other works [118]. The third stage of degradation was observed from 300 to 500 °C, which can be attributed to the carbon oxidative decomposition, initiating the carbonization process indicated by DTG at the greatest temperature degradation at ~345 °C. As expected, the hydrogels did not show significant differences in mass loss because only the nanocellulose concentration was modified. Figure S5 shows the thermograms of all hydrogel samples and the components used for their synthesis. Overall, the hydrogels are stable up to approximately 200 °C; this property confirms that these materials have the required thermal stability for use as wound dressings or, where appropriate, for steam sterilization [133,134].

### 3.10. Mechanical Studies of Hydrogels

**Figure 15:**
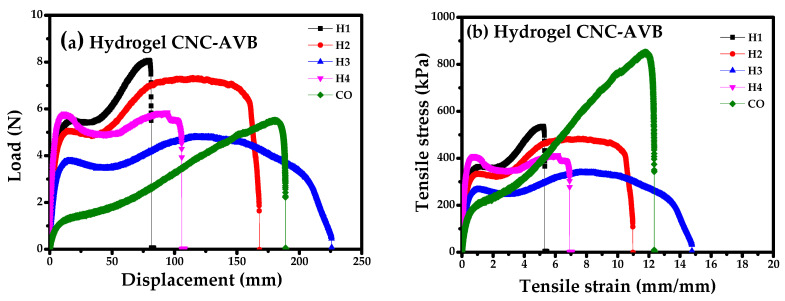

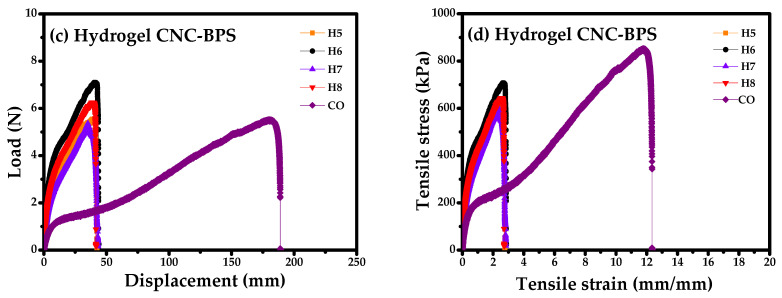
Load–displacement and stress–strain curves for hydrogels obtained from (**a**,**b**) CNC-AVB and (**c**,**d**) CNC-BPS.

Figure 15a–d show the stress–strain curves under tension of the hydrogel samples labeled as H1 to H8. Figure 15a,b for CNC-AVB hydrogels (H1, H2, H3, and H4) show higher tensile strain at break compared to CNC-BPS hydrogels (H5, H6, H7, and H8) (Figure 15c,d), obtaining the highest tensile strain at a maximum load in H3 of 14.78 mm/mm, i.e., 1478% of percent elongation at break or ductility, and a tensile stress at a maximum load (tensile strength) of 341.30 kPa. On the other hand, CNC-BPS hydrogels (H5, H6, H7, and H8) present greater tensile stress at maximum load, with specimen H6 being the one that exhibits the greatest tensile stress at maximum load or tensile strength of 707.67 kPa; therefore, CNC-AVB shows a higher percent elongation but lower tensile strength. On the contrary, the highest tensile strength observed in the hydrogels is found in CNC-BPS, which is attributed to the fact that they present a higher percentage of crystallinity compared to CNC-AVB (see also Table 6). The characteristics of nanocellulose crystals make them a promising candidate for reinforcement in the synthesis of hydrogels as they improve mechanical strength.

It has been previously stated that relative humidity negatively influences Young’s modulus, but this effect depends on the nature and structural state of the plant cell wall polymers. At the same time, relative humidity can promote adhesion between CNC and other polymers; therefore, its adequate control is a key factor for achieving optimal mechanical properties [135]. In this case, the studies were realized with a controlled *RH* of 73%, meaning that the results are only dependent on the synthesis parameters and biomass type.

CNC can be physically mixed into the polymeric hydrogel matrix in small concentrations ranging from 0.1 to 5 wt% of the total weight of the hydrogel [136]. When used via chemical action, high-performance nanocomposites are obtained; covalently cross-linked CNC present versatile properties, stability, and potential for medical applications. However, it has been reported that chemical cross-linking produces stronger hydrogels but with lower swelling and water absorption [136]. The hydrogels were compared to a commercial hydrocolloid dressing Duoderm™ Extra Thin Spots. This is a 0.52 mm thick simple protective dressing for the treatment of mild abrasions and dry or slightly exudative superficial wounds. It has an inner film of three hydrocolloids (gelatin, pectin, and sodium carboxymethylcellulose). The outer film is composed of polyurethane foam that gives it greater mechanical properties and is obtained synthetically. However, hydrogels based on cellulose, chitosan, and citric acid, synthesized from natural polymers, are competitive alternatives because they achieve a higher percent elongation compared to commercial ones.

## 4. Conclusions

Despite the significant advances in the development of new materials for biomedical applications, the functionality of CNC in isolation is limited. In this study, CNC was separated from aloe vera bagasse (AVB) and banana pseudostem (BPS) waste to evaluate the dependence of crystallinity on the source and synthesis parameters and their influence on overall hydrogel performance for functional biomedical materials. From the wide variety of characterizations performed at different steps, the following results were obtained: it was found that the parameters that directly impacted hydrogel crystallinity, and, consequently, the overall performance, were the temperature and reaction time, while less impact was observed from the acid concentration. The size of CNC remained stable, with a polydispersity degree that varied between 0.20 and 0.71. The results of cellulose-based hydrogels derived from these wastes indicated that, by modulating the crystallinity through the careful control of synthesis parameters, it can be enhanced or balanced with crucial parameters required for materials used in biomedical applications, such as porosity, adsorption capacity, thermal stability, and mechanical properties. Therefore, the CNC isolated from AVB and BPS can effectively enhance hydrogel performance, supporting the viability of using green materials for developing eco-friendly alternatives in the biomedical field. However, our research group still needs to address certain action lines to highlight their final application, following on from the current research. First, biocompatibility, adhesion tests, and hydrogel degradation should be studied. Regarding the improvement or generation of properties such as antibacterial, anti-inflammatory, and antioxidant properties, nanoparticles have been incorporated to generate a bacteria-inhibiting material and improve its resistance properties. One issue that is still not fully understood is the healing mechanism during the multiple stages that take place. In general, consideration of these points is required for a complete study and to ensure its application in the biomedical field.

## Figures and Tables

**Table 1:** Hydrogen bond energies and distances in nanocellulose crystals for the aloe vera bagasse.

Source AVB	Group Assignment O_6_H·O_3_ 3100–3310 cm^−1^			Group Assignment O_3_H·O_5_ 3310–3340 cm^−1^			Group Assignment O_2_H·O_6_ 3340–3500 cm^−1^			Group Assignment Free OH Groups OH (2) 3560–3513 cm^−1^ OH (6) 3577–3579 cm^−1^		
	Band Position (cm^−1^)	EH (kJ/mol)	R (Å)	Band Position (cm^−1^)	EH (kJ/mol)	R (Å)	Band Position (cm^−1^)	EH (kJ/mol)	R (Å)	Band Position (cm^−1^)	EH (kJ/mol)	R (Å)
C_45_t_30_T_25_	3142	36.53	2.73	3312	24.58	2.77	3470	13.09	2.81	3571	5.74	2.83
C_45_t_30_T_40_	3149	36.03	2.73	3314	24.44	2.77	3472	12.94	2.81	3568	5.96	2.83
C_45_t_30_T_45_	3140	36.67	2.73	3319	24.07	2.77	3477	12.58	2.81	3570	5.81	2.83
C_45_t_30_T_50_	3151	35.88	2.73	3325	23.64	2.77	3478	12.51	2.81	3570	5.81	2.83
C_45_t_60_T_25_	3134	37.11	2.73	3320	24.00	2.77	3477	12.58	2.81	3564	6.25	2.83
C_45_t_60_T_40_	3127	37.61	2.73	3320	24.00	2.77	3464	13.53	2.80	3549	7.34	2.82
C_45_t_60_T_45_	3139	36.75	2.73	3322	23.85	2.77	3485	12.00	2.81	3572	5.67	2.83
C_45_t_60_T_50_	3135	37.03	2.73	-	-	-	3445	14.91	2.80	3545	7.63	2.82
	3297	25.67	2.77									
C_55_t_30_T_25_	3159	35.31	2.74	3333	23.05	2.78	3493	11.42	2.81	3570	5.81	2.83
C_55_t_30_T_40_	-	-	-	3327	23.22	2.77	3395	18.54	2.79	3562	6.40	2.83
							3495	11.27	2.81			
C_55_t_30_T_45_	3115	38.47	2.73	-	-	-	3425	16.36	2.80	3552	7.12	2.82
	3284	26.62	2.76	-	-	-						
C_55_t_30_T_50_	3224	30.63	2.75	3394	18.62	2.79	3511	10.11	2.82	3579	5.16	2.83
C_55_t_60_T_25_	3145	36.31	2.73				3442	15.13	2.80	3569	5.89	2.83
	3294	25.89	2.77									
C_55_t_60_T_40_	3155	35.59	2.74	3324	23.71	2.77	3477	12.58	2.81	3561	6.44	2.83
C_55_t_60_T_45_	3153	35.74	2.73	3328	23.42	2.77	3479	12.43	2.81	3561	6.47	2.83
C_55_t_60_T_50_	3145	36.31	2.73	3339	22.62	2.78	3443	15.05	2.80	3568	5.96	2.83
	3299	25.53	2.77									

**Table 2:** Hydrogen bond energies and distances in nanocellulose crystals from banana pseudostem.

Source BPS	Group Assignment O_6_H·O_3_ 3100–3310 cm^−1^			Group Assignment O_3_H·O_5_ 3310–3340 cm^−1^			Group Assignment O_2_H·O_6_ 3340–3500 cm^−1^			Group Assignment Free OH Groups OH (2) 3560–3513 cm^−1^ OH (6) 3577–3600 cm^−1^		
	Band Position (cm^−1^)	EH (kJ/mol)	R (Å)	Band Position (cm^−1^)	EH (kJ/mol)	R (Å)	Band Position (cm^−1^)	EH (kJ/mol)	R (Å)	Band Position (cm^−1^)	EH (kJ/mol)	R (Å)
C_45_t_30_T_25_	3183	33.58	2.74	3339	22.62	2.781	3354	21.53	2.78	3549	7.34	2.82
	3275	27.27	2.76									
C_45_t_30_T_40_	3153	35.74	2.73				3341	22.47	2.78	3554	6.98	2.75
	3292	26.04	2.77				3426	16.29	2.80			
							3493	11.42	2.81			
C_45_t_30_T_45_	3140	36.67	2.73	3337	22.76	2.78	3440	15.27	2.80	3565	6.18	2.83
	3282	26.76	2.76									
C_45_t_30_T_50_	3154	35.67	2.73				3340	22.54	2.78	3564	6.25	2.83
	3280	26.91	2.76				3401	18.11	2.79			
C_45_t_60_T_25_	3138	36.82	2.73	3339	22.62	2.78	3427	16.22	2.80	3571	5.74	2.83
	3283	26.69	2.76									
C_45_t_60_T_40_	3130	37.39	2.73	3339	22.62	2.78	3429	16.07	2.80	3545	7.63	2.82
	3281	26.84	2.76									
C_45_t_60_T_45_	3144	36.39	2.73	3327	23.49	2.77	3440	15.27	2.80	3526	9.02	2.82
							3492	11.49	2.81			
C_45_t_60_T_50_	3138	36.82	2.73				3341	22.47	2.78	3572	5.67	2.83
	3299	25.53	2.77				3458	13.96	2.80			
C_55_t_30_T_25_	3138	36.82	2.73	3312	24.58	2.77	3461	13.74	2.80	3543	7.78	2.82
C_55_t_30_T_40_	3173	34.30	2.74	-	-	-	3346	22.11	2.78	3519	9.52	2.75
	3304	25.16	2.77				3383	19.42	2.79			
							3441	15.20	2.80			
C_55_t_30_T_45_	3153	35.74	2.73	-	-	-	3354	21.53	2.78	3522	9.31	2.82
	3300	25.45	2.77				3445	14.91	2.80	3596	3.92	2.75
C_55_t_30_T_50_	3155	35.59	2.74	-	-	-	3350	21.82	2.78	3524	9.16	2.82
	3292	26.04	2.77				3435	15.63	2.80	3591	4.29	2.75
C_55_t_60_T_25_	3136	36.96	2.73	-	-	-	3340	22.54	2.78	3568	5.96	2.83
	3299	25.53	2.77				3450	14.54	2.80			
C_55_t_60_T_40_	3145	36.31	2.73	3339	22.62	2.78	3462	13.67	2.80	3560	6.54	2.83
	3309	24.80	2.77									
C_55_t_60_T_45_	3145	36.31	2.73	3323	23.78	2.77	3460	13.82	2.80	3545	7.63	2.82
C_55_t_60_T_50_	3140	36.67	2.73	3338	22.69	2.78	3453	14.33	2.80	3565	6.18	2.83
	3296	25.75	2.77									

**Table 3:** Yield obtained during the different acid hydrolysis treatments for the extraction of nanocellulose crystals: (a) CNC-AVB and (b) CNC-BPS.

(a)		(b)	
Source CNC-AVB	Nanocellulose Yield (%)	Source CNC-BPS	Nanocellulose Yield (%)
C_45_t_30_T_25_	47.63 ± 1.29	C_45_t_30_T_25_	57.22 ± 1.45
C_45_t_30_T_40_	47.57 ± 0.03	C_45_t_30_T_40_	57.99 ± 2.81
C_45_t_30_T_45_	49.90 ± 5.86	C_45_t_30_T_45_	59.21 ± 2.50
C_45_t_30_T_50_	48.65 ± 5.1	C_45_t_30_T_50_	58.75 ± 1.04
C_45_t_60_T_25_	48.72 ± 5.13	C_45_t_60_T_25_	57.2 ± 1.29
C_45_t_60_T_40_	49.65 ± 5.21	C_45_t_60_T_40_	51.98 ± 9.77
C_45_t_60_T_45_	48.47 ± 1.19	C_45_t_60_T_45_	50.03 ± 6.40
C_45_t_60_T_50_	48.77 ± 4.98	C_45_t_60_T_50_	49.22 ± 5.70
C_55_t_30_T_25_	46.61 ± 1.27	C_55_t_30_T_25_	54.31 ± 2.41
C_55_t_30_T_40_	48.85 ± 1.99	C_55_t_30_T_40_	56.79 ± 5.60
C_55_t_30_T_45_	48.50 ± 5.19	C_55_t_30_T_45_	58.26 ± 2.09
C_55_t_30_T_50_	49.60 ± 2.73	C_55_t_30_T_50_	58.78 ± 0.94
C_55_t_60_T_25_	49.61 ± 4.79	C_55_t_60_T_25_	55.18 ± 0.03
C_55_t_60_T_40_	45.06 ± 9.01	C_55_t_60_T_40_	55.8 ± 0.20
C_55_t_60_T_45_	46.19 ± 0.92	C_55_t_60_T_45_	56.82 ± 0.49
C_55_t_60_T_50_	46.65 ± 1.30	C_55_t_60_T_50_	57.54 ± 0.54

**Table 4:** Crystallinity index, crystal size, and peak index from deconvolution data obtained for different acid hydrolysis treatments.

	Nanocellulose from BPS					Nanocellulose from AVB				
	Cellulose Iα	Cellulose Iβ	Cellulose II	*C.S.* (nm)	*CI* (*%*)	Cellulose Iα	Cellulose Iβ	Cellulose II	*C.S.* (nm)	*CI* (%)
C_45_t_30_T_25_	15.42°$\left(0\bar{1}1\right)$ 20.22°$\left(\bar{1}\bar{1}2\right)$ 21.80°$\left(\bar{1}10\right)$	13.80°(011) 14.85°$\left(\bar{1}10\right)$ 16.66°(110) 22.98°(200)	12.26°$\left(100\right)$ 19.92°$\left(110\right)$	3.65	73.13	14.26°$\left(100\right)$ 5.24°$\left(0\bar{1}1\right)$ 18.65°$\left(\bar{1}\bar{1}0\right)$ 21.80°$(\bar{1}10$)	13.8°$\;\left(011\right)$ 16.6° $\left(110\right)$	4.94°$\left(101\right)$ 19.77°$\left(113\right)$	30.70	88.97
C_45_t_30_T_40_				37.20	72.47				27.20	72.68
C_45_t_30_T_45_				31.50	76.12				34.00	88.88
C_45_t_30_T_50_				33.10	87.76				27.50	89.66
C_45_t_60_T_25_	15.42°$\left(0\bar{1}1\right)$ 20.22°$\left(\bar{1}\bar{1}2\right)$ 21.80°$\left(\bar{1}10\right)$	14.27°(101) 14.85°$\left(\bar{1}10\right)$ 16.66°(110) 22.98°(200)	12.26°$\left(100\right)$ 17.18°(002) 19.92°$\left(\bar{1}20\right)$ 21.58°(110)	36.30	83.92	15.42°$\left(0\bar{1}1\right)$ 20.63°$\left(002\right)$	13.80°$\left(011\right)$ 14.85°$\left(\bar{1}10\right)$ 16.66°$\left(110\right)$ 20.27°$\left(012\right)$, 22.98°$\left(200\right)$	21.13°$\left(102\right)$	32.00	85.19
C_45_t_60_T_40_				43.70	93.6				35.70	88.33
C_45_t_60_T_45_				42.40	86.79				28.10	73.13
C_45_t_60_T_50_				40.20	81.47				28.70	70.67
C_55_t_30_T_25_	15.42°$\left(0\bar{1}1\right)$ 20.22°$\left(\bar{1}\bar{1}2\right)$ 21.80°$\left(\bar{1}10\right)$	13.80°(011), 14.85°$\left(\bar{1}10\right)$ 16.66°(110) 22.98°(200)	12.26°$\left(100\right)$ 19.92°$\left(110\right)$	33.10	77.86	15.42°$\left(0\bar{1}1\right)$ 21.80°$\;\left(\bar{1}10\right)$	13.80°(011) 14.27°(101) 16.66°(110)	17.18°(002) 19.77°$\left(\bar{1}20\right)$ 19.92°(110)	31.10	88.52
C_55_t_30_T_40_				33.50	76.33				33.40	88.01
C_55_t_30_T_45_				23.40	87.64				34.90	81.74
C_55_t_30_T_50_				31.20	97.58				33.60	78.91
C_55_t_60_T_25_	15.42°$\left(0\bar{1}1\right)$ 18.65°$\left(\bar{1}\bar{1}1\right)$	13.80°(011) 14.85°$\left(\bar{1}10\right)$ 16.66°(110) 22.98°(200)	12.26°$\left(100\right)$ 19.92°$\left(110\right)$	38.50	79.66	20.22° $\left(\bar{1}\bar{1}2\right)$ 21.80°$\;\left(\bar{1}10\right)$	13.80°(011) 14.27°(101) 14.85°$\left(\bar{1}10\right)$ 18.74°(111) 22.98°(200)	12.26°(100) 14.94°(101) 17.18°(002) 19.77°$\left(\bar{1}20\right)$ 21.58°$\left(\bar{1}21\right)$	36.20	83.93
C_55_t_60_T_40_				68.30	88.41				32.20	82.64
C_55_t_60_T_45_				43.20	88.47				37.50	73.08
C_55_t_60_T_50_				49.10	87.46				39.10	86.07

**Table 5:** Hydrogel physicochemical profile of the prepared samples from AVB and BPS.

Hydrogel	Swelling % (pH 4 and 12 h)	Gel (%)	Porosity (%)	Pore Size (μm)
H1	202.01	81.91 ± 2.86	53.45 ± 1.24	2.38 ± 0.39
H2	217.50	79.13 ± 1.42	55.09 ± 0.90	2.26 ± 0.39
H3	210.67	78.19 ± 1.85	54.89 ± 0.67	2.53 ± 0.55
H4	206.72	77.93 ± 1.88	53.37 ± 6.28	3.15 ± 0.10
H5	208.63	84.26 ± 1.81	58.20 ± 1.46	2.52 ± 0.87
H6	215.26	85.94 ± 1.86	60.77 ± 2.6	2.60 ± 0.34
H7	225.39	83.45 ± 2.67	58.80 ± 0.97	2.81 ± 0.28
H8	218.68	86.60 ± 2.62	57.37 ± 0.86	2.45 ± 0.11

**Table 6:** Mechanical performance of hydrogels.

Hydrogel	Maximum Load (N)	Extension at Maximum Load (mm)	Extension at Break (mm)	Tensile Stress at Maximum Load (kPa)	Tensile Strain at Maximum Load (mm)	Tensile Strain at Break (mm/mm)
H1	8.08	79.45	83.45	537.15	5.19	5.45
H2	7.33	114.75	168.25	484.74	7.50	11.00
H3	4.82	117.35	226.10	341.30	7.67	14.78
H4	5.86	94.20	108.03	413.87	6.16	7.06
H5	5.55	39.87	42.63	615.06	2.61	2.79
H6	7.10	40.13	43.98	707.67	2.62	2.88
H7	5.36	35.20	43.33	609.40	2.30	2.83
H8	6.25	38.30	41.93	644.17	2.50	2.74
Commercial (CO)	5.52	179.82	189.33	852.97	11.75	12.38

## Data Availability

All the data are reported in this study.

## Supplementary Materials

The following supporting information can be downloaded at: https://www.mdpi.com/article/10.3390/polym17121642/s1. References [137,138,139,140,141,142,143] are cited in the Supplementary Materials. Table S1. FTIR analysis of hydrogen bonding using AVB as source (second derivative analysis); Table S2. FTIR analysis of hydrogen bonding using BPS as source (second derivative analysis); Table S3. Relative content of three types of hydrogen bonds in aloe vera bagasse nanocellulose crystals and free OH supplementary; Table S4. Relative content of three types of hydrogen bonds in banana pseudo steam nanocellulose crystals and free OH; Table S5. Comparison NC yield of various raw materials and hydrolysis conditions; Table S6. Comparison hydrogel swelling capability and fraction gel; Figure S1. Deconvolution of nanocellulose samples treated with H_2_SO_4_ for nanocellulose (45 and 55 wt.%), temperature (25, 40, 45 and 50 °C), reaction time (30 and 60 min): (a) NC-AVB (nanocellulose from aloe vera bagasse)/45 wt.%/30 min; (b) NC-AVB/45 wt.%/60 min, (c) NC-AVB/55 wt.%/30 min, (d) NC-AVB/55 wt.%/60 min; Figure S2. Deconvolution of nanocellulose samples treated with H_2_SO_4_ for nanocellulose (45 and 55 wt.%), temperature (25, 40, 45 and 50 °C), reaction time (30 and 60 min): (a) NC-BPS (nanocellulose from BPS)/45 wt.%/30 min; (b) NC-BPS/45 wt.%/60 min, (c) NC-BPS/55 wt.%/30 min, (d) NC-BPS/55 wt.%/60 min.
