# Supplementary material for: Engineering to Improve Mechanical Properties of Nanocellulose Hydrogels from Aloe Vera Bagasse and Banana Pseudostem for Biomedical Applications
Source: Polymers (Basel). 2025 Jun 13;17(12):1642. doi: 10.3390/polym17121642 (PMC12196950; doi:10.3390/polym17121642)
Supplement: Supplementary file 1 [file polymers-17-01642-s001.zip › polymers-3643139-supplementary.pdf]

Supporting information for:

Table S1. FTIR analysis of hydrogen bonding using AVB as source (second derivative analysis)

Source NANOCELLULOSE AVB	Center Max	group assignment	Center Max	group assignment	Center Max	group assignment	Center Max	group assignment	Center Max	group assignment	Center Max	group assignment
**$C_{45}t_{30}T_{25}$**	3142	O_6-H···$O_{3'}$ (celulosa I)	3312	O_3-H···O_5	3470	O_2-H···O_6	3571	Free OH Groups	------	------	------	------
**$C_{45}t_{30}T_{40}$**	3149	O_6-H···$O_{3'}$	3314	O_3-H···O_5	3472	O_2-H···O_6	3568	Free OH Groups	------	------	------	------
**$C_{45}t_{30}T_{45}$**	3140	O_6-H···$O_{3'}$	3319	O_3-H···O_5	3477	O_2-H···O_6	3570	Free OH Groups	------	------	------	------
**$C_{45}t_{30}T_{50}$**	3151	O_6-H···$O_{3'}$	3325	O_3-H···O_5	3478	O_2-H···O_6	3570	Free OH Groups	------	------	------	------
**$C_{45}t_{60}T_{25}$**	3134	O_6-H···$O_{3'}$	3320	O_3-H···O_5	3477	O_2-H···O_6	3564		------	------	------	------
**$C_{45}t_{60}T_{40}$**	3127	O_6-H···$O_{3'}$	3320	O_3-H···O_5	3464	O_2-H···O_6	3549		------	------	------	------
**$C_{45}t_{60}T_{45}$**	3139	O_6-H···$O_{3'}$	3322	O_3-H···O_5	3485	O_2-H···O_6	3572	Free OH Groups	------	------	------	------
**$C_{45}t_{60}T_{50}$**	3135	O_6-H···$O_{3'}$	3297	O_6-H···O_3	3445	O_2-H···O_6	3545		------	------	------	------
**$C_{55}t_{30}T_{25}$**	3159	O_6-H···$O_{3'}$	3333	O_3-H···O_5	3493	O_2-H···O_6	3570	Free OH Groups	------	------	------	------
**$C_{55}t_{30}T_{40}$**	3327	O_6-H···$O_{3'}$	3395	O_3-H···O_5	3495	O_2-H···O_6	3562		------	------	------	------
**$C_{55}t_{30}T_{45}$**	3115	O_6-H···$O_{3'}$	3284	O_6-H···O_3	3425	O_2-H···O_6	3552		------	------	------	------
**$C_{55}t_{30}T_{50}$**	3224	O_6-H···$O_{3'}$	3394	O_3-H···O_5	3511	-----	3579	Free OH Groups	------	------	------	------
**$C_{55}t_{60}T_{25}$**	3145	O_6-H···$O_{3'}$	3294	O_6-H···O_3	3442	O_2-H···O_6	3569	Free OH Groups	------	------	------	------
**$C_{55}t_{60}T_{40}$**	3155	O_6-H···$O_{3'}$	3324	O_3-H···O_5	3477	O_2-H···O_6	3561		------	------	------	------
**$C_{55}t_{60}T_{45}$**	3153	O_6-H···$O_{3'}$	3328	O_3-H···O_5	3479	O_2-H···O_6	3561		------	------	------	------
**$C_{55}t_{60}T_{50}$**	3145	O_6-H···$O_{3'}$	3299	O_3-H···O_5	3339	O_3-H···O_5	3443	O_2-H···O_6	3568			------

Table S2. FTIR analysis of hydrogen bonding using BPS as source (second derivative analysis)

Source NANOCELLULOSE BPS	Center Max	group assignment	Center Max	group assignment	Center Max	group assignment	Center Max	group assignment	Center Max	group assignment	Center Max	group assignment
**$C_{45}t_{30}T_{25}$**	3183	O_6-H···$O_{3'}$	3275	O_6-H···O_3	3339	O_3-H···O_5	3354	O_3-H···O_5	3549		------	------
**$C_{45}t_{30}T_{40}$**	3153	O_6-H···$O_{3'}$	3292	O_6-H···O_3	3341	O_3-H···O_5	3426	O_2-H···O_6	3493	Free OH Groups	3554	------
**$C_{45}t_{30}T_{45}$**	3140	O_6-H···$O_{3'}$	3282	O_6-H···O_3	3337	O_3-H···O_5	3440	O_2-H···O_6	3565	Free OH Groups	------	------
**$C_{45}t_{30}T_{50}$**	3154	O_6-H···$O_{3'}$	3280	O_6-H···O_3	3340	O_3-H···O_5	3401	O_2-H···O_6	3564	Free OH Groups	------	------
**$C_{45}t_{60}T_{25}$**	3138	O_6-H···$O_{3'}$	3283	O_6-H···O_3	3339	O_3-H···O_5	3427	O_2-H···O_6	3571	Free OH Groups	------	------
**$C_{45}t_{60}T_{40}$**	3130	O_6-H···$O_{3'}$	3281	O_6-H···O_3	3339	O_3-H···O_5	3429	O_2-H···O_6	3545		------	------
**$C_{45}t_{60}T_{45}$**	3144	O_6-H···$O_{3'}$	3327	O_3-H···O_5	3440	O_2-H···O_6	3492	O_2-H···O_6	3526		------	------
**$C_{45}t_{60}T_{50}$**	3138	O_6-H···$O_{3'}$	3299	O_6-H···O_3	3341	O_3-H···O_5	3458	O_2-H···O_6	3572	Free OH Groups	------	------
**$C_{55}t_{30}T_{25}$**	3138	O_6-H···$O_{3'}$	3312	O_3-H···O_5	3461	O_2-H···O_6	3543		------	------	------	------
**$C_{55}t_{30}T_{40}$**	3173	O_6-H···$O_{3'}$	3304	O_3-H···O_5	3346	O_3-H···O_5	3383	O_3-H···O_5	3441	O_2-H···O_6	3519	
**$C_{55}t_{30}T_{45}$**	3153	O_6-H···$O_{3'}$	3300	O_3-H···O_5	3354	O_3-H···O_5	3445	O_2-H···O_6	3522		3596	Free OH Groups
**$C_{55}t_{30}T_{50}$**	3155	O_6-H···$O_{3'}$	3292	O_6-H···O_3	3350	O_3-H···O_5	3435	O_2-H···O_6	3524		3591	Free OH Groups
**$C_{55}t_{60}T_{25}$**	3136	O_6-H···$O_{3'}$	3299	O_6-H···O_3	3340	O_3-H···O_5	3450	O_2-H···O_6	3568	Free OH Groups	------	------
**$C_{55}t_{60}T_{40}$**	3145	O_6-H···$O_{3'}$	3309	O_3-H···O_5	3339	O_3-H···O_5	3462	O_2-H···O_6	3560	Free OH Groups	------	------
**$C_{55}t_{60}T_{45}$**	3145	O_6-H···$O_{3'}$	3323	O_3-H···O_5	3460	O_2-H···O_6	3545		------	------	------	------
**$C_{55}t_{60}T_{50}$**	3140	O_6-H···$O_{3'}$	3296	O_6-H···O_3	3338	O_3-H···O_5	3453	O_3-H···O_5	3565	Free OH Groups	------	------

Table S3. Relative content of three types of hydrogen bonds in aloe vera bagasse nanocellulose crystals and free OH supplementary

SOURCE AVB	GROUP ASSIGNMENT O_6-H···$O_{3'}$ 3100-3310 CM^{-1}		GROUP ASSIGNMENT O_3-H···O_5 3310-3340 CM^{-1}		GROUP ASSIGNMENT O_2-H···O_6 3340-3500 CM^{-1}		GROUP ASSIGNMENT FREE OH GROUPS OH (2) 3560-3513 CM^{-1} OH (6) 3577-3579 CM^{-1}	
	Fitted peak center/ cm^{-1}	Area ratio %	Fitted peak center/ cm^{-1}	Area ratio %	Fitted peak center/ cm^{-1}	Area ratio %	Fitted peak center/ cm^{-1}	Area ratio %
$C_{45}T_{30}T_{25}$	3142	8.847	3312	67.057	3470	22.152	3571	1.944
$C_{45}T_{30}T_{40}$	3149	8.595	3314	68.089	3472	20.540	3568	2.776
$C_{45}T_{30}T_{45}$	3140	8.243	3319	76.634	3477	12.285	3570	2.837
$C_{45}T_{30}T_{50}$	3151	10.578	3325	70.993	3478	14.948	3570	3.482
$C_{45}T_{60}T_{25}$	3134	7.755	3320	78.031	3477	11.768	3564	2.445
$C_{45}T_{60}T_{40}$	3127	7.477	3320	80.843	3464	6.438	3549	5.242
$C_{45}T_{60}T_{45}$	3139	8.796	3322	75.834	3485	12.270	3572	3.100
$C_{45}T_{60}T_{50}$	3135<br>3297	8.451<br>55.825			3445	27.986	3545	7.739
$C_{55}T_{30}T_{25}$	3159	11.303	3333	74.043	3493	10.940	3570	3.714
$C_{55}T_{30}T_{40}$			3327	91.443	3395<br>3495	1.559<br>6.057	3562	0.941
$C_{55}T_{30}T_{45}$	3115	2.561			3425	36.839	3552	5.204
	3284	55.396						
$C_{55}T_{30}T_{50}$	3224	35.042	3394	52.139	3511	10.268	3579	2.550
$C_{55}T_{60}T_{25}$	3145<br>3294	10.416<br>52.016			3442	36.256	3569	1.312
$C_{55}T_{60}T_{40}$	3155	8.958	3324	69.047	3477	18.162	3561	3.833
$C_{55}T_{60}T_{45}$	3153	10.010	3328	70.337	3479	15.911	3561	3.742
$C_{55}T_{60}T_{50}$	3145<br>3299	14.849<br>54.939	3339	0.643	3443	28.878	3568	0.691

Table S4. Relative content of three types of hydrogen bonds in banana pseudo steam nanocellulose crystals and free OH

SOURCE BPS	GROUP ASSIGNMENT O_6-H···$O_{3'}$ 3100-3310 CM^{-1}		GROUP ASSIGNMENT O_3-H···O_5 3310-3340 CM^{-1}		GROUP ASSIGNMENT O_2-H···O_6 3340-3500 CM^{-1}		GROUP ASSIGNMENT FREE OH GROUPS OH (2) 3560-3513 CM^{-1} OH (6) 3577-3579 CM^{-1}	
	Fitted peak center/ cm^{-1}	Area ratio %	Fitted peak center/ cm^{-1}	Area ratio %	Fitted peak center/ cm^{-1}	Area ratio %	Fitted peak center/ cm^{-1}	Area ratio %
**$C_{45}T_{30}T_{25}$**	3183	16.192	3339	0.922	3354	76.521	3549	2.799
	3275	3.567						
**$C_{45}T_{30}T_{40}$**	3153	15.376			3341	0.879	3554	2.924
	3292	49.115			3426	31.460		
					3493	0.245		
**$C_{45}T_{30}T_{45}$**	3140	7.019	3337	0.902	3440	35.113	3565	0.703
	3282	56.262						
**$C_{45}T_{30}T_{50}$**	3154	13.101			3340	1.052	3564	1.475
	3280	36.471			3401	47.901		
**$C_{45}T_{60}T_{25}$**	3138	12.399	3339	0.462	3427	36.762	3571	1.393
	3283	48.985						
**$C_{45}T_{60}T_{40}$**	3130	8.006	3339	0.361	3429	31.430	3545	9.463
	3281	50.740						
**$C_{45}T_{60}T_{45}$**	3144	10.147	3327	76.551	3440	4.144	3526	8.356
					3492	0.802		
**$C_{45}T_{60}T_{50}$**	3138	9.581			3341	0.526	3572	1.685
	3299	59.863			3458	28.344		
**$C_{55}T_{30}T_{25}$**	3138	6.065	3312	81.165	3461	10.747	3543	2.023
**$C_{55}T_{30}T_{40}$**	3173	21.898			3346	2.488	3519	6.728
	3304	38.374			3383	4.533	3592	2.357
					3441	23.622		
**$C_{55}T_{30}T_{45}$**	3153	20.020			3354	1.428	3522	2.906
	3300	34.879			3445	36.934	3596	3.833
**$C_{55}T_{30}T_{50}$**	3155	16.760			3350	1.459	3524	2.945
	3292	38.715			3435	38.554	3591	1.567
**$C_{55}T_{60}T_{25}$**	3136	11.059			3340	0.481	3568	0.623
	3299	64.267			3450	23.569		
**$C_{55}T_{60}T_{40}$**	3145	7.079	3339	0.669	3462	24.358	3560	3.257
	3309	64.636						
**$C_{55}T_{60}T_{45}$**	3145	12.391	3323	75.698	3460	8.940	3545	2.971
**$C_{55}T_{60}T_{50}$**	3140	8.266	3338	0.628	3453	29.456	3565	1.879
	3296	59.770						

Table S5. Comparison NC yield of various raw materials and hydrolysis conditions.

Raw materials	% Yield	Hydrolysis conditions	References
Aloe vera bagasse	49.9 ± 5.86	H_2SO_4 45% (w/w), 45 °C for 30 min	This work
Banana pseudostem	59.21 ± 2.5	H_2SO_4 45% (w/w), 45 °C for 30 min	This work
Royal palm tree	42.4	H_2SO_4 64% (w/w), 45 °C for 20 min	[1]
Corn cob	50.07	H_2SO_4 30 % (w/w), 50 °C for 30 min	[2]
Wheat bran	37.1	H_2SO_4 64% (w/w), 45°C for 30 min	[3]
Bamboo fiber	22	H_2SO_4 64% (v/v), 45 °C for 45 min	[4]
Grape pomace	12	H_2O_2 (w/w), 50 °C for 4 hours	[5]
Cocoa pod husk	25	H_2SO_4 64 % (w/v), 45 °C for 60 min	[6]

Table S6. Comparison hydrogel swelling capability and fraction gel.

Hydrogel	Swelling equilibrium % (pH 4 and 12 h)	Gel (%)	Porosity (%)	Pore size (µm)	Reference
H1 (hydrogel NC-AVB $C_{45}t_{30}T_{50}$)	202.01	81.91 ± 2.86	53.45 ± 1.24	2.38 ± 0.39	This work
H2 ($C_{45}t_{60}T_{40}$)	217.50	79.13 ± 1.42	55.09 ± 0.90	2.26 ± 0.39	This work
H3 ($C_{55}t_{30}T_{50}$)	210.67	78.19 ± 1.85	54.89 ± 0.67	2.53 ± 0.55	This work
H4 ($C_{55}t_{60}T_{45}$)	206.72	77.93 ± 1.88	53.37 ± 6.28	3.15 ± 0.10	This work
H5 (hydrogels NC- BPS $C_{45}t_{30}T_{50}$)	208.63	84.26 ± 1.81	58.20 ± 1.46	2.52 ± 0.87	This work
H6 ($C_{45}t_{60}T_{40}$)	215.26	85.94 ± 1.86	60.77 ± 2.6	2.60 ± 0.34	This work
H7 ($C_{55}t_{30}T_{25}$)	225.39954	83.45 ± 2.67	58.80 ± 0.97	2.81 ± 0.28	This work
H8 ($C_{55}t_{60}T_{50}$)	218.68172	86.60 ± 2.62	57.37 ± 0.86	2.45 ± 0.11	This work
OHA-DA-PAM/CMP/TGF-β1 composite hydrogel	---	---	---	70-120	[7]
Nanocellulose/wood ash-reinforced starch chitosan hydrogel composites	~400	65 - 88	76-68	---	[8]
Silver nanoparticles loaded cellulose-acetate based multifunctional dressing	200	---	90.52 ± 2.42	---	[9]
Bacterial Cellulose-Based Wound Dressing with impregnation of Chitosan and Collagen	99.6 ± 0,04	---	70.9 ± 0,10	---	[10]
Asymmetric natural wound dressing based on porous chitosan-alginate hydrogel/electrospun PCL-silk sericin loaded by 10-HDA	175	---	---	519 (nm)	[11]
Hydrogel based in polymer N-carboxyethyl chitosan (CEC) and oxidized hyaluronic acid-graft-aniline tetramer (OHA-AT) polymer	36	---	---	19.5 - 36.7	[12]

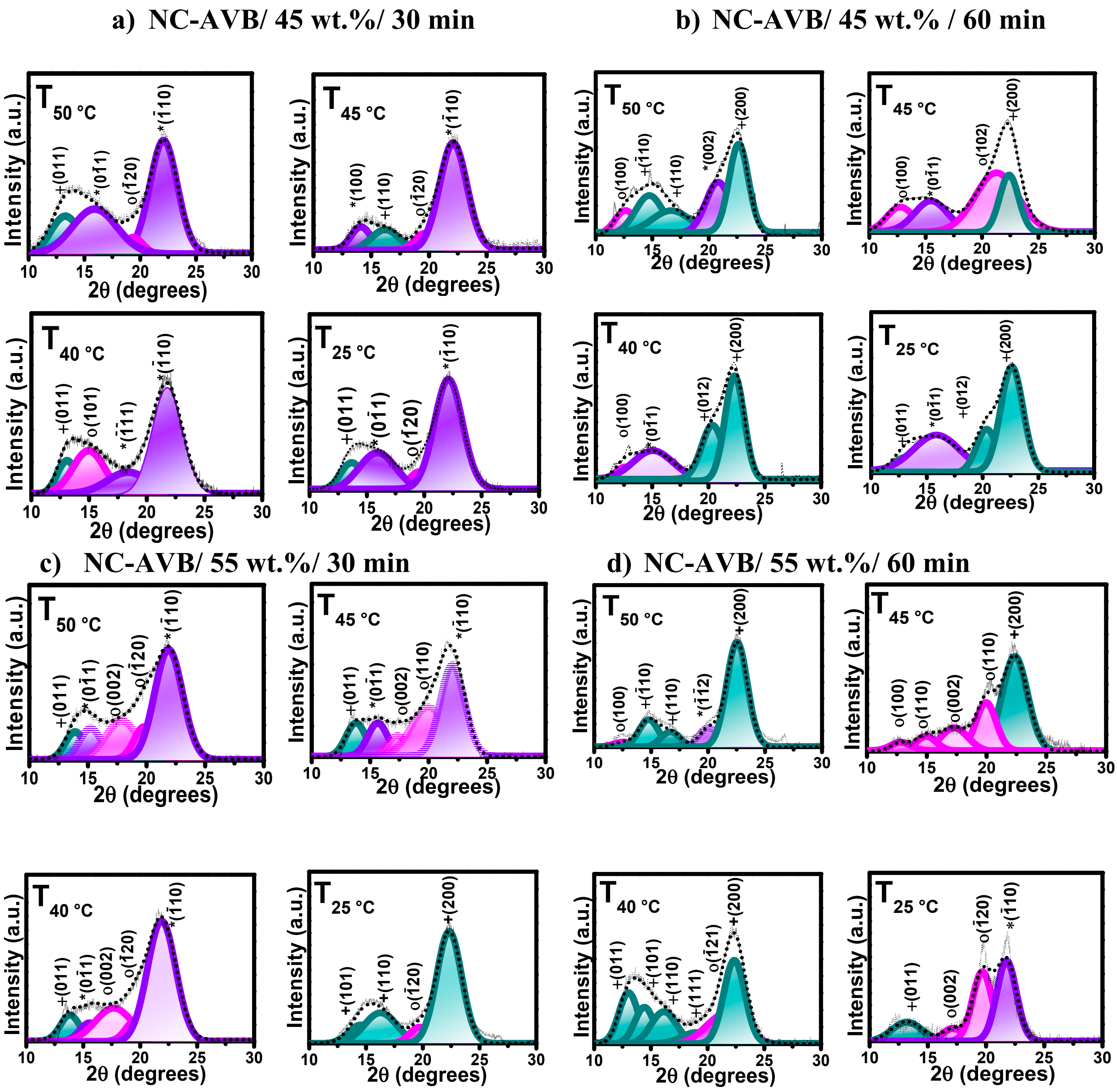

Figure S1. Deconvolution of nanocellulose samples treated with H_2SO_4 for nanocellulose (45 and 55 wt.%), temperature (25, 40, 45 and 50 °C), reaction time (30 and 60 min): (a) NC-AVB (nanocellulose from aloe vera bagasse) / 45 wt.%/ 30 min; (b) NC-AVB/ 45 wt.% / 60 min, (c) NC-AVB/ 55 wt.% / 30 min, (d) NC-AVB/ 55 wt.% / 60 min.

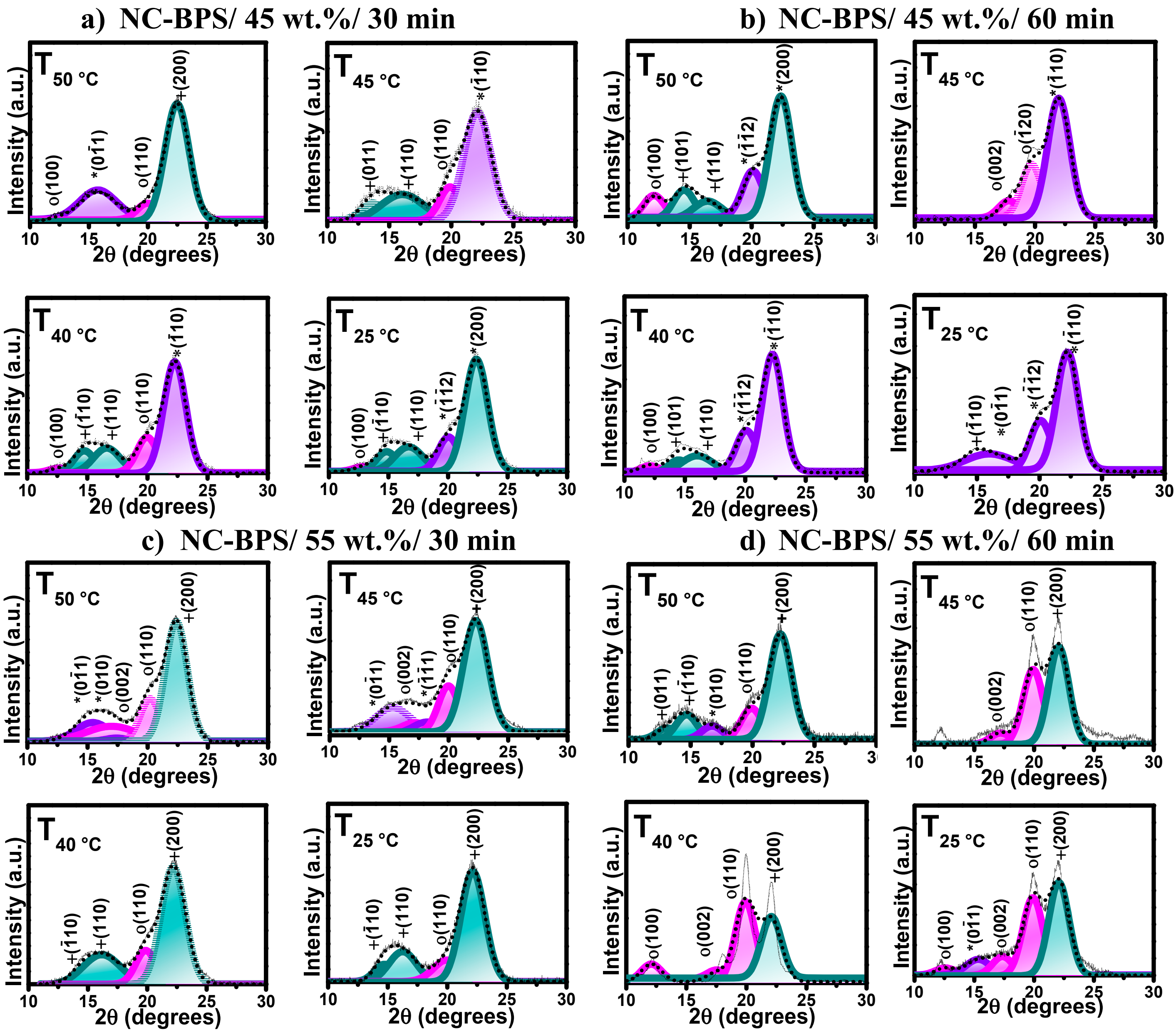

Figure S2. Deconvolution of nanocellulose samples treated with H_2SO_4 for nanocellulose (45 and 55 wt.%), temperature (25, 40, 45 and 50 °C), reaction time (30 and 60 min): (a) NC-BPS (nanocellulose from BPS) / 45 wt.%/ 30 min; (b) NC-BPS/ 45 wt.% / 60 min, (c) NC-BPS/ 55 wt.% / 30 min, (d) NC-BPS/ 55 wt.% / 60 min.

References

[1] E. Hafemann, R. Battisti, C. Marangoni, R.A.F. Machado, Valorization of royal palm tree agroindustrial waste by isolating cellulose nanocrystals, Carbohydrate Polymers 218 (2019) 188-198.https://doi.org/10.1016/j.carbpol.2019.04.086.
[2] D. Sartika, A.P. Firmansyah, I. Junais, I.W. Arnata, F. Fahma, A. Firmanda, High yield production of nanocrystalline cellulose from corn cob through a chemical-mechanical treatment under mild conditions, International Journal of Biological Macromolecules 240 (2023) 124327.https://doi.org/10.1016/j.ijbiomac.2023.124327.
[3] Y. Xiao, Y. Liu, X. Wang, M. Li, H. Lei, H. Xu, Cellulose nanocrystals prepared from wheat bran: Characterization and cytotoxicity assessment, International Journal of Biological Macromolecules 140 (2019) 225-233.https://doi.org/10.1016/j.ijbiomac.2019.08.160.
[4] M. Rasheed, M. Jawaid, B. Parveez, A. Zuriyati, A. Khan, Morphological, chemical and thermal analysis of cellulose nanocrystals extracted from bamboo fibre, International Journal of Biological Macromolecules 160 (2020) 183-191.https://doi.org/10.1016/j.ijbiomac.2020.05.170.
[5] C.C.d.S. Coelho, R.B.S. Silva, C.W.P. Carvalho, A.L. Rossi, J.A. Teixeira, O. Freitas-Silva, L.M.C. Cabral, Cellulose nanocrystals from grape pomace and their use for the development of starch-based nanocomposite films, International Journal of Biological Macromolecules 159 (2020) 1048-1061.https://doi.org/10.1016/j.ijbiomac.2020.05.046.
[6] A.I. Akinjokun, L.F. Petrik, A.O. Ogunfowokan, J. Ajao, T.V. Ojumu, Isolation and characterization of nanocrystalline cellulose from cocoa pod husk (CPH) biomass wastes, Heliyon 7(4) (2021) e06680.10.1016/j.heliyon.2021.e06680.
[7] Z. Wei, H. Ye, Y. Li, X. Li, Y. Liu, Y. Chen, J. Yu, J. Wang, X. Ye, Mechanically tough, adhesive, self-healing hydrogel promotes annulus fibrosus repair via autologous cell recruitment and microenvironment regulation, Acta Biomaterialia 178 (2024) 50-67.https://doi.org/10.1016/j.actbio.2024.02.020.
[8] D.N. Iqbal, Z. Tariq, B. Philips, A. Sadiqa, M. Ahmad, K.M. Al-Ahmary, I. Ali, M. Ahmed, Nanocellulose/wood ash-reinforced starch–chitosan hydrogel composites for soil conditioning and their impact on pea plant growth, RSC Advances 14(13) (2024) 8652-8664.10.1039/D3RA08725E.
[9] S. Dugam, R. Jain, P. Dandekar, Silver nanoparticles loaded triple-layered cellulose-acetate based multifunctional dressing for wound healing, International Journal of Biological Macromolecules 276 (2024) 133837.https://doi.org/10.1016/j.ijbiomac.2024.133837.
[10] K.M. Pasaribu, S. Gea, S. Ilyas, T. Tamrin, I. Radecka, Characterization of Bacterial Cellulose-Based Wound Dressing in Different Order Impregnation of Chitosan and Collagen, in: (Ed.)^(Eds.) Biomolecules, 2020, p.^pp. 10.3390/biom10111511.
[11] M. Nazemoroaia, F. Bagheri, S.Z. Mirahmadi-Zare, F. Eslami-kaliji, A. Derakhshan, Asymmetric natural wound dressing based on porous chitosan-alginate hydrogel/electrospun PCL-silk sericin loaded by 10-HDA for skin wound healing: In vitro and in vivo studies, International Journal of Pharmaceutics 668 (2025) 124976.https://doi.org/10.1016/j.ijpharm.2024.124976.
[12] J. Qu, X. Zhao, Y. Liang, Y. Xu, P.X. Ma, B. Guo, Degradable conductive injectable hydrogels as novel antibacterial, anti-oxidant wound dressings for wound healing, Chemical Engineering Journal 362 (2019) 548-560.https://doi.org/10.1016/j.cej.2019.01.028.
